# TIP60 acts as a regulator of genes involved in filopodia formation and cell migration during wound healing

**DOI:** 10.1016/j.jbc.2022.102015

**Published:** 2022-05-05

**Authors:** Shraddha Dubey, Bharti Jaiswal, Ashish Gupta

**Affiliations:** 1 Epigenetics and Human Disease Laboratory, Department of Life Sciences, Shiv Nadar University, Uttar Pradesh, India; 2 Integrative Chemical Biology, Institute for Stem Cell Science and Regenerative Medicine (inStem), Bangalore, India

**Keywords:** TIP60, PXR, filopodia, wound healing, cell migration, histone acetylation, gene regulation, nuclear receptor, CD, chromodomain, DBD, DNA-binding domain, Ni-NTA, Ni-nitrilotriacetic acid

## Abstract

Wound healing is a complex phenomenon that requires coordination of numerous molecular and cellular changes to facilitate timely and efficient repair of the damaged tissue. Although many of these molecular pathways have been detailed, others remain to be elucidated. In the present work, we show for the first time, roles for the acetyltransferase TIP60 and nuclear receptor transcription factor PXR in this process, participating in wound healing by altering actin dynamics and cellular motility. We found that in response to wound-injury, TIP60 induces rapid formation of filopodia at the wounded cell front, leading to enhanced cell migration and faster closure of the wound. Further, qPCR analysis revealed heightened expression of *Cdc42* and *ROCK1* genes, key regulators involved in filopodia formation and actin reorganization, exclusively in TIP60-PXR-expressing cells upon wound-induction. We also performed ChIP assays to confirm the context-specific binding of TIP60 on the *ROCK1* promoter and demonstrated that the TIP60 chromodomain is essential for loading of the TIP60–PXR complex onto the chromatin. Results from immunoprecipitation assays revealed that during the wounded condition, TIP60 alters the chromatin microenvironment by specifically acetylating histones H2B and H4, thereby modulating the expression of target genes. Overall, findings of this study show that TIP60 is a novel regulator of the wound healing process by regulating the expression of wound repair-related genes.

Despite decades of research, our understanding of the cellular and molecular mechanisms underpinning the wound-recovery process are still poorly understood. The entire process of wound healing is highly complicated and is characterized by a series of precisely regulated events that occur in different phases: inflammatory, proliferative, and the tissue remodeling phase (1, 2, 3, 4). A number of biochemical, molecular, and cellular changes takes place around the wound site that facilitates timely and efficient repair of the damaged tissue. Among various cellular processes that are needed to heal an injury, cell migration is one of the key mechanisms that not only helps inflammatory cells to reach the wounded site but also enables local cells near the wound area to promote re-epithelialization, a necessary step to complete the wound healing process (5, 6, 7, 8). Controlled cell migration is crucial for proper wound repair, as aberrant cell migration can cause delayed wound healing and can contribute to development of chronic non-healing wound, hypertrophic, and keloid scars and may also increase the risk of developing cancer (9, 10, 11). Altered migratory activities of cells due to various factors like aging, medicinal drugs, and chronic diseases like diabetes mellitus and vascular diseases can also impact the body's ability to heal wounds (12, 13, 14, 15, 16, 17).

Dynamic reorganization of actin cytoskeleton is absolutely essential for inducing the morphological changes in the cell required to achieve transition from a motionless state to a motile phenotype. A diverse array of actin-based protrusion structures like filopodia and lamellipodia formed in the cell membrane are phenotypic indicators of migrating cell and also determine the polarization and directional movement of the cell (18, 19, 20, 21). The cytoskeletal dynamics and reorganization is guided by coordinated actions of different cellular factors and signaling pathways that helps to adopt the formation of three-dimensional assemblies of actin protrusions depending on the cellular conditions (22, 23).

Recently, we showed PXR (a well-established member of nuclear receptor superfamily) as a novel interacting partner of TIP60 (an essential lysine acetyl transferase protein) and identified their role in cell migration and adhesion (24, 25). However, the mechanisms utilized by this newly identified complex underpinning enhanced cell migratory activities, and the physiological context in which this complex becomes functional, remained unidentified. In this present study, we have for the first time demonstrated that TIP60-PXR initiate rapid and enhanced formation of filopodia in response to wound stimuli that enhance the rate of cell migration and promote faster wound healing. More specifically, we have also identified the gene targets through screening and evaluation of candidate genes related to filopodia formation, cell migration, and wound healing, in presence of TIP60-PXR under wound-generated condition. Interestingly, the RT-qPCR result revealed that in response to wound stimuli, TIP60-PXR induces expression of *Cdc42, ROCK1, IGFBP-1*, and *Gadd45β* genes. Through ChIP analysis, we confirmed the context-dependent binding of TIP60 on promoter of one of these genes, *ROCK1*. Further analysis revealed that TIP60 specifically acetylates histone H2B and H4 during wound-induced condition, leading to TIP60-PXR mediated alterations in chromatin environment, accountable for elevated expression of wound repair-related genes.

## Results

### TIP60-PXR induces rapid formation of filopodia resulting in faster wound closure

**Figure 1:**
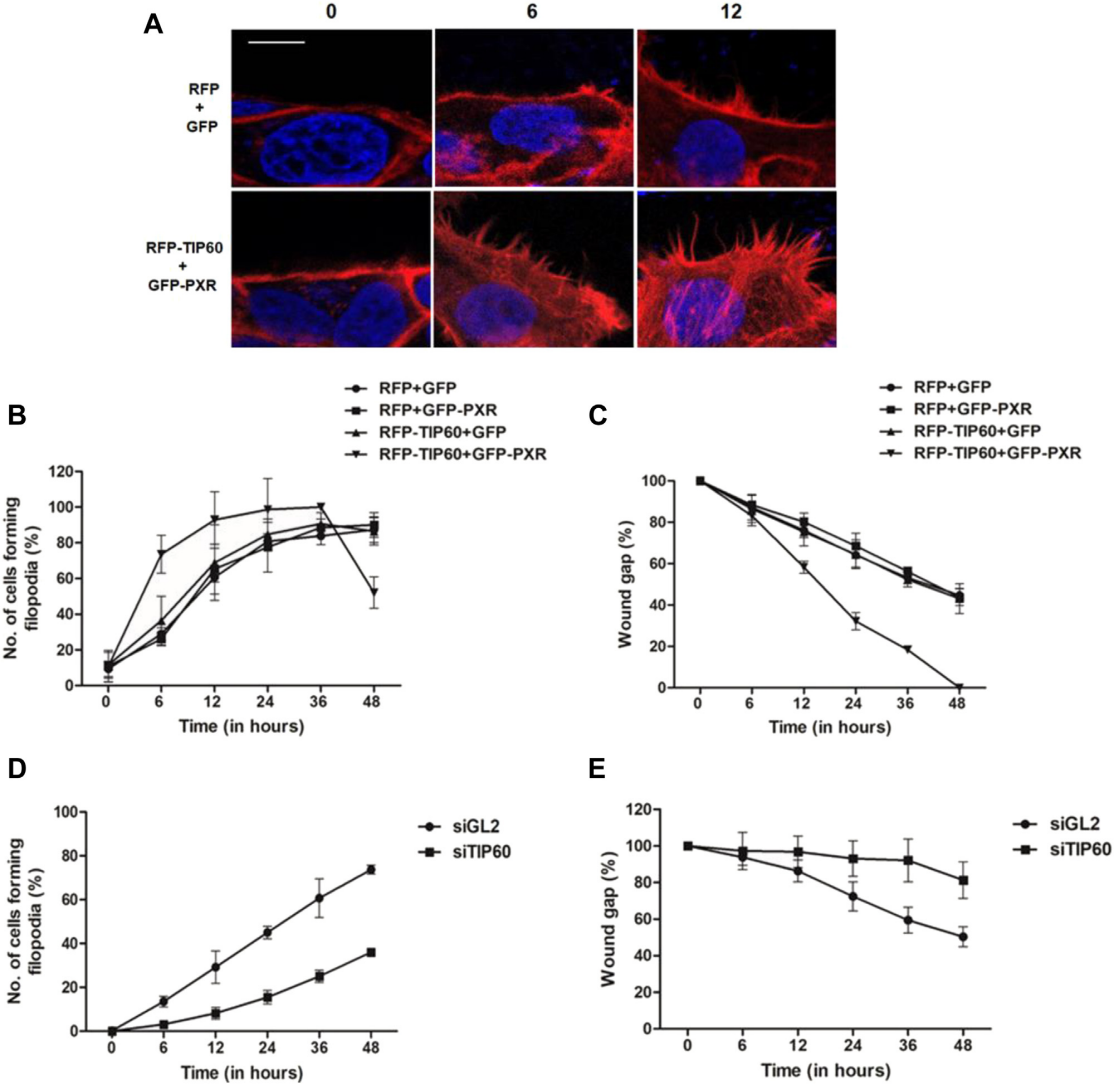
**TIP60–PXR complex induces rapid filopodia formation and faster wound closure.***A*, TIP60-PXR promotes early filopodia formation. Wound was generated in confluent monolayer of HepG2 cells transiently transfected with indicated plasmid combinations and microscopy was performed to visualize the formation of filopodia at different time points after staining the cells with fluorescently labeled phalloidin (*red color*). Nucleus was stained with DAPI (*blue color*). Bar is equivalent to 10 μm. *B*, number of cells forming filopodia post wound induction, at different time points (0, 6, 12, 24, 36, and 48 h) in HepG2 cells transfected with indicated plasmid combinations. Graph depicts percent average value (with ±S.D.). *p* values (for RFP-GFP *versus* RFP-TIP60+GFP-PXR) at 6 and 48 h are 0.0185 and 0.0213, respectively. *C*, wound gap filling correlates with TIP60-PXR induced filopodia formation. *In vitro* wound healing scratch assay was performed with HepG2 cells transfected with plasmids as indicated in the figure, and wound gap filling was monitored at different time points post wound generation, and graph was plotted for the average values of three independent experimental replicates with ±S.D. *p* values (for RFP-GFP+ *versus* RFP-TIP60+GFP-PXR) at 12, 24, 36, and 48 h were calculated to be 0.0018, 0.0002, <0.0001, and <0.0001, respectively. *D*, TIP60 knockdown inhibit filopodia formation. HepG2 cells were transfected with siRNA oligos against TIP60 or GL2 (control), and filopodia formation was monitored at different time points as indicated. Graph shows percentage average value of three independent experiments with ±S.D. *p* values for siGL2 vs siTIP60 at 6, 12, 24, 36, and 48 h are 0.0020, 0.0097, 0.0003, 0.0026, and <0.0001 respectively. *E*, TIP60 knockdown impedes wound healing. HepG2 cells were transfected with siTIP60 or siGL2 (control) oligos, and scratch was generated in confluent monolayer of cells. Wound gap filling was monitored at different time points (0, 6, 12, 24, 36, and 48 h) post wound induction. Graph depicts percentage average value (with ±S.D.) of three independent experimental replicates at 0, 6, 12, 24, 36, and 48 h post scratch generation. *p* values for siGL2 *versus* siTIP60 at 24, 36, and 48 h were calculated to be 0.0453, 0.0146, and 0.0089, respectively.

The fact that TIP60-PXR overexpression induce significant enhancement in the cellular motility, we wanted to determine whether TIP60–PXR complex stimulates the formation of actin-based protrusions like filopodia and lamellipodia, some of the well-characterized phenotypic features of the migrating cells. To examine this, *in vitro* wound was generated in HepG2 cells cotransfected with TIP60 and PXR, and the morphology of the cell was examined for formation of actin protrusions. After staining with Alexa Fluor 594 phalloidin, microscopic analysis of the wound area showed clear formation of thin needle-like actin-rich membrane extensions (characteristic feature of filopodia), at the leading edge of the migrating cells, and the maturation of the filopodia was accompanied by increase in their size (Fig. 1*A*). Most importantly, time course analysis for filopodia formation revealed, early and rapid formation of filopodia, as early as 6 h of wounding and in relatively more number of cells expressing TIP60 and PXR when compared with control cells, where it took double the time for filopodia formation to be detected (Fig. 1*B*). Next, to examine the effect of early formation of filopodia on the rate of wound gap closure, *in vitro* wound healing assay was performed and wound gap filling was monitored at regular time intervals from 0 to 48 h. The results showed noticeable difference in the gap filling from 6 h onward in cells coexpressing TIP60 and PXR, which correlated positively with timing of filopodia formation. The difference increasingly became more conspicuous by 12 h, and the gap was completely filled within 48 h in cells coexpressing TIP60 and PXR (Fig. 1*C*). Wound filling could not be completed in any other control set even after 48 h had passed. The graph shows the percentage gap filling of the wound area plotted over time at regular time intervals (Fig. 1*C*). We got similar results in Huh-7 cells, where the cells coexpressing TIP60 and PXR showed early filopodia formation and wound gap closure (Fig. S1, *A* and *B*).

Given that TIP60 induce intranuclear organization of PXR and TIP60-PXR overexpression induce filopodia formation and cell migration, we wanted to examine the effect of TIP60 knockdown on these processes during wound-generated condition. For this, siRNA-mediated knockdown of TIP60 was performed in HepG2 cells (Fig. S3), and filopodia formation was observed at different time points. Result showed drastic reduction in filopodia formation in TIP60-depleted conditions compared to control starting from 6 h onward (Fig. 1*D*). Time course analysis for wound gap closure under similar conditions revealed severe impact on HepG2 cells capacity to fill the wound gap (Fig. 1*E*).

Together, these results show that TIP60–PXR complex plays critical role in promoting wound healing process by inducing rapid filopodia formation and cell migration.

### TIP60–PXR complex induce expression of genes involved in actin reorganization and filopodia formation

**Figure 2:**
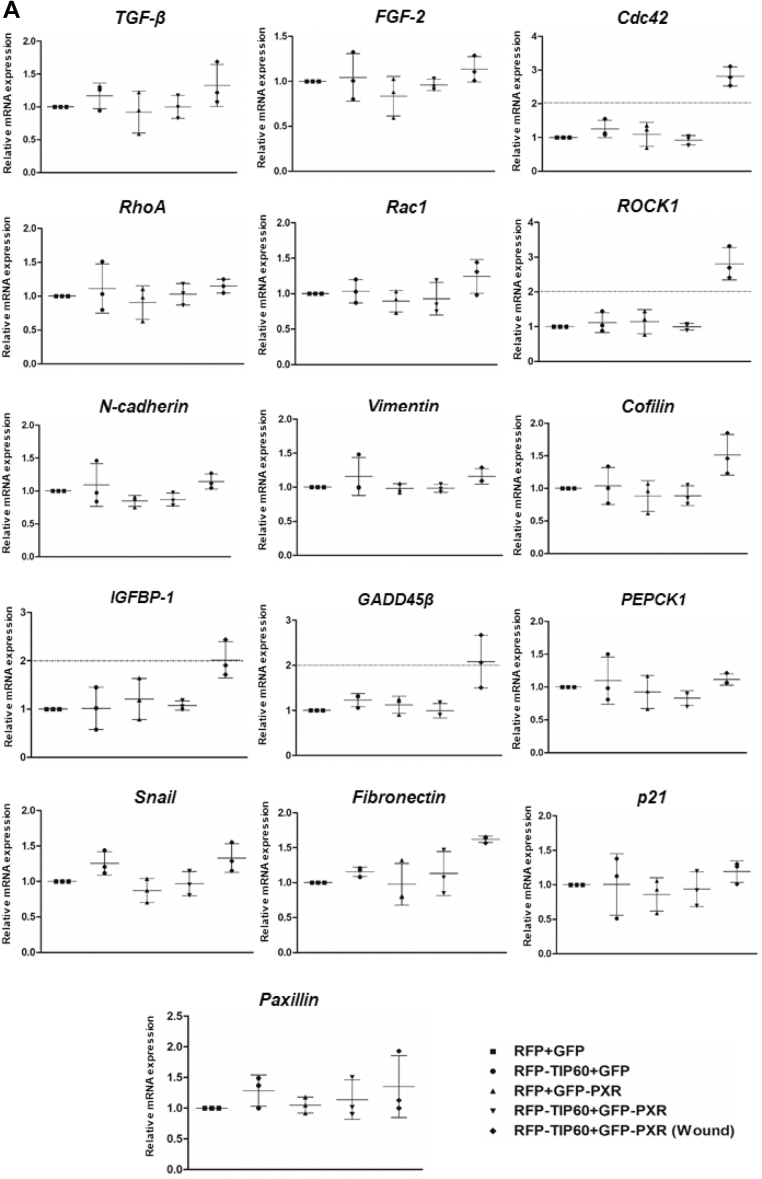

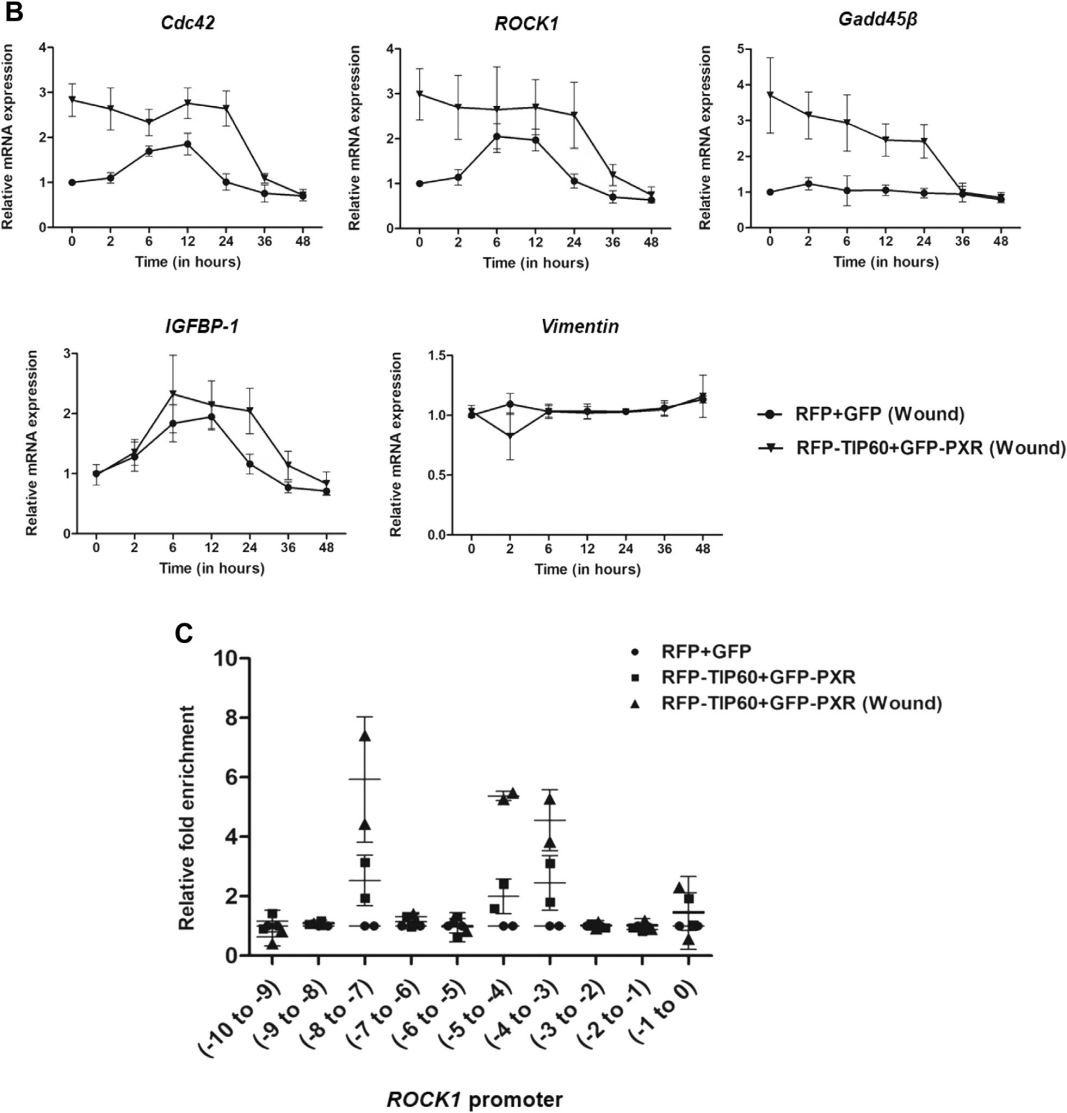
**TIP60–PXR complex upregulates expression of *Cdc42, ROCK1, IGFBP-1*, and *Gadd45β* in response to wound generation.***A*, RT-qPCR analysis was performed with transfected HepG2 cells to analyze the expression of candidate genes in response to wounding, at 24 h of wound generation. Scatter plots depict the relative gene expression of candidate genes by taking value of control set, RFP+GFP as 1. *GAPDH* was used as an internal control. Average value of three independent experiments was calculated with ±S.D. Y-axis shows relative fold induction in mRNA levels. *p* values (for RFP+GFP *versus* RFP-TIP60+GFP-PXR (wound)) for *Cdc42, ROCK1, IGFBP-1*, and *GADD45β* are <0.0001, <0.0001, 0.0147, and 0.0050, respectively. *B*, time-course analysis of TIP60-PXR dependent upregulated gene expression by RT-qPCR analysis. To examine the time-dependent expression of upregulated genes, RT-qPCR was performed with harvested HepG2 cell extracts at different time points after wound generation. Graph depicts average mean value of three independent experimental replicates with ±S.D. *p* values for *Cdc42* at 2 and 24 h time-points is 0.0053 and 0.0028, for *ROCK1* at 2 and 24 h is 0.0217 and 0.0277, for *IGFBP-1* at 24 h is 0.0207, and for *GADD45β* at 2 and 24 h is 0.0080 and 0.0064, respectively. *C*, detection of TIP60 occupancy on *ROCK1* gene promoter by ChIP-qPCR analysis under wound-generated condition. Using TIP60 antibody, ChIP-assay was performed with transfected HepG2 cell extracts under control or wound-generated conditions. Subsequently, qPCR analysis was performed to examine the binding of TIP60 on *ROCK1* promoter, using series of primers, each covering 1 kb region from 10 kb upstream to transcription start site (-10 to -9, -9 to-8, -8 to -7, -7 to -6, -6 to -5, -5 to -4, -4 to -3, -3 to -2, -2 to -1, -1 to 0). Scatter plot depicts average mean value of two independent experiments (with ±S.D.) showing relative enrichment of TIP60 occupancy on *ROCK1* promoter. *p* values (for RFP+GFP versus RFP-TIP60+GFP-PXR (wound)) for TIP60 occupancy at -5 to -4 & -4 to -3 region was 0.0022 and 0.0458, respectively.

After observing the effect of TIP60 and PXR on rapid filopodia formation and faster wound closure, we wanted to identify the potential downstream gene targets that could be modulated by TIP60-PXR in response to wounding. For this, we performed real-time PCR with the harvested cell extracts coexpressing TIP60 and PXR during wound-generated conditions, to examine the expression level of some of the important genes linked with filopodia formation, cell migration, and wound healing (Table S1). Among various genes that were examined for their mRNA expression level, we observed heightened expression of *Cdc42, ROCK1, IGFBP-1*, and *Gadd45β* genes only in cells coexpressing TIP60 and PXR, under wound-generated state (Fig. 2*A*). To confirm the reliability of our qPCR data, we examined *Cdc42* and *ROCK1* gene expression using two more internal controls (*β-actin* and *28S* rRNA) and the result showed similar increase (as observed with *GAPDH* as internal control) in the expression of these genes in wound-induced condition showing robustness of our data (Fig. S2, *A* and *B*).

In order to map the changes in the expression level of these genes, from the time right after wounding the cells till complete filling of the wound gap, we performed real-time PCR analysis with the cell extract harvested at 0, 2, 6, 12, 24, 36, and 48 h under wound-generated state. Interestingly, we found instant escalation of *Cdc42, ROCK1*, and *Gadd45β* genes right after wounding, which remained elevated till 24 h (Fig. 2*B*). On the contrary, a gradual increase in *IGFBP-1* expression was observed which peaked at 6 h. The expression of all these genes started declining after 24 h of wounding. The expression level of *Vimentin* used as a negative control for the experiment remained unaffected for the duration of the experiment.

Next, to examine whether TIP60–PXR complex binds directly to the promoter of their targeted genes, we performed ChIP assay on *ROCK1* gene promoter up to 10 kbp upstream region to examine and define the binding regions of TIP60. Results showed binding of TIP60 on *ROCK1* gene promoter at -8 to -7 and -5 to -3 upstream regions during wound-generated conditions (Fig. 2*C*). Overall, these findings demonstrate context-dependent binding of TIP60-PXR on their targeted gene promoters that causes enhanced expression of genes triggering rapid formation of filopodia and enhanced cell migration required for faster wound closure.

### TIP60–PXR complex acetylates histone H2B and H4 during wound healing

**Figure 3:**
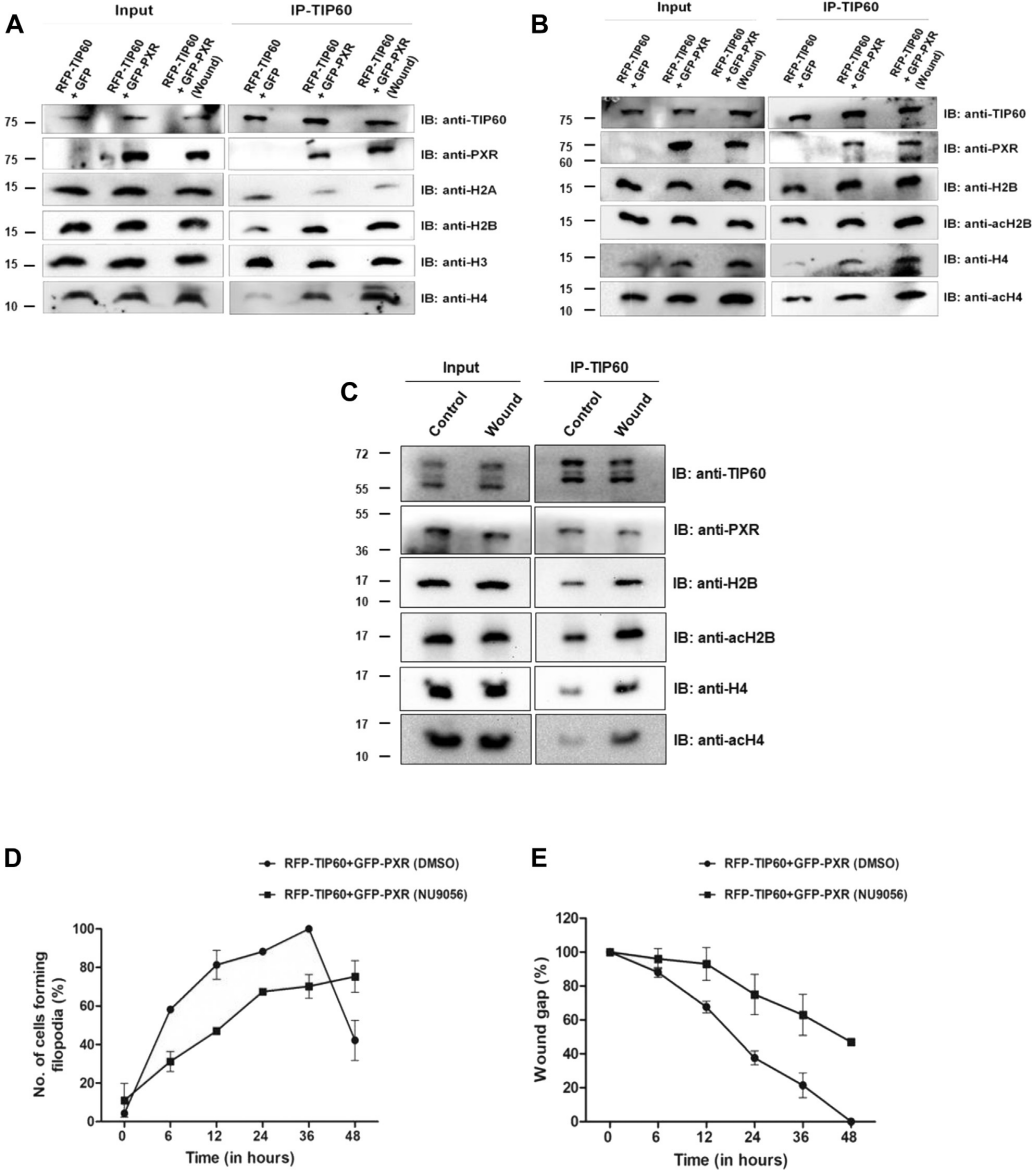
**TIP60 catalytic activity is required for TIP60**-**PXRmediated histone H2B and H4 acetylation, filopodia formation, and wound gap closure.***A*, TIP60-PXR interacts with H2B & H4 during wound generated condition. Immunoprecipitation of TIP60 was performed from Cos-1 cells transfected with indicated plasmid combinations under control or wound-generated conditions. Western blot analysis was performed with immunoprecipitated samples with TIP60, PXR, & core histone antibodies as indicated in the figure. *B*, TIP60-PXR acetylates H2B & H4 during wound-generated condition. Immunoprecipitation experiment using TIP60 antibody was performed in similar manner as described in (A) followed by Western blot analysis of the immunoprecipitated samples with indicated antibodies in the figure. *C*, endogenous TIP60–PXR complex acetylates H2B & H4 under wound-generated conditions. *In vitro* scratch was generated in completely confluent HepG2 cells and after 24 h of wound generation, endogenous TIP60–PXR complex was immunoprecipitated using TIP60 antibody and Western blot analysis of pulled down endogenous proteins were performed with mentioned antibodies. All full-length blot images are provided in Fig. S4. *D*, inhibition of TIP60 catalytic activity reduces filopodia formation. *In vitro* wound scratch was generated in HepG2 cells transfected with RFP-TIP60 and GFP-PXR plasmids, followed by treatment with DMSO or NU9056. Number of cells forming filopodia at the wound edge were counted at 0, 6, 12, 24, 36, and 48 h time-point. Graph was plotted for the average number of cells (%) forming filopodia obtained with two independent experimental replicates performed in duplicates. *p* values for 6, 12, and 24 h time-points are 0.0188, 0.0230, and 0.0027, respectively. *E*, inhibition of TIP60 catalytic activity impairs wound gap filling ability of HepG2 cells. *In vitro* wound scratch assay was performed as described in (C) followed by treatment with DMSO or NU9056. Filling of wound gap was monitored at different time points as indicated and graph was plotted for the average mean value of three independent experimental replicates. *p* values for 12, 24, and 36 h time-points are 0.0129, 0.0067, and 0.0071, respectively.

Since TIP60 is a chromatin modifier protein and is known to acetylate histones at various lysine residues, we wanted to examine the interaction of TIP60–PXR complex with different core histones and the resulting outcome of these interactions on acetylation status of core histone proteins during wound-generated condition. For this, we performed TIP60 immunoprecipitation assay to determine TIP60–PXR complex interaction with various core histones under control and wound-generated conditions. Western blot result showed that TIP60 was successfully immunoprecipitated in all the samples (Fig. 3*A*). PXR was also pulled down along with TIP60 in samples expressing TIP60 and PXR in both normal and wound-generated conditions. Under similar conditions, all the four core histones were pulled down along with TIP60; however, we observed enhanced interaction of TIP60 with histone H2B and H4 under wound-generated condition (Fig. 3*A*). After observing the context-specific interaction of TIP60–PXR complex with H2B and H4, we wanted to examine the effect of TIP60–PXR complex on acetylation levels of H2B and H4 histones under similar conditions. To examine this, we performed TIP60 immunoprecipitation assay as described above and performed Western blot analysis with acetylated H2B and H4 antibodies. Results showed significantly enhanced acetylation level of histone H2B and H4 under wound-generated condition (Fig. 3*B*). Further to confirm the observations, we performed immunoprecipitation of endogenous TIP60 protein from HepG2 cells followed by Western blot analysis. We found similar results showing enhanced interaction of TIP60–PXR complex with both core histones H2B and H4 and also observed significantly enhanced acetylation levels of these histones during wound-generated condition (Fig. 3*C*).

After identifying TIP60-PXR complex–dependent acetylation of histones during wound-generated condition, we wanted to determine the effect of TIP60 catalytic HAT activity on filopodia formation and wound-repair activity. For this, we performed *in vitro* wounding assay with confluent monolayer of cells coexpressing TIP60 and PXR in presence of NU9056 (TIP60 inhibitor) or DMSO (control), and the cells forming filopodia were counted at different time intervals till 48 h. Interestingly, we observed significant reduction in number of cells forming filopodia in presence of NU9056 (Fig. 3*D*). Moreover, when we examined the effect of NU9056 on wound healing capacity of these cells, we found that compromising the catalytic activity of TIP60 results in impaired wound gap healing ability of NU9056-treated cells (Fig. 3*E*).

Altogether, these results demonstrate that TIP60–PXR complex acetylates H2B and H4 histones under wound-generated conditions and catalytic HAT activity of TIP60 is crucial in controlling filopodia formation and wound healing capacity of cells.

### TIP60 chromodomain is indispensable for loading of TIP60–PXR complex onto the chromatin and its wound healing function

**Figure 4:**
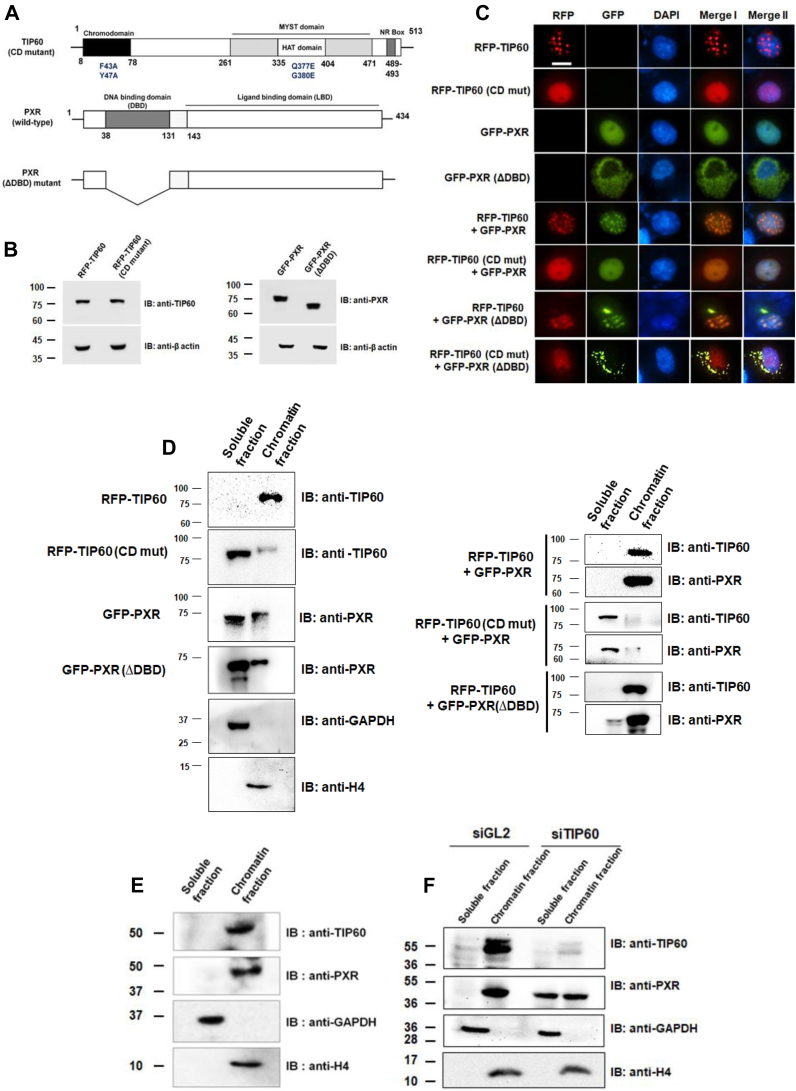
**TIP60 chromodomain is essential for loading of TIP60–PXR complex onto the chromatin.***A*, schematic diagram representing point mutations generated in TIP60 chromodomain (CD) and DBD deletion (ΔDBD) in PXR. *B*, Western blots showing the expression of RFP-TIP60 (WT), RFP-TIP60 (CD mutant), GFP-PXR (WT), and GFP-PXR (ΔDBD) constructs at their expected size. *C*, TIP60 CD domain is important for forming TIP60 & PXR colocalized nuclear foci. Live cell imaging was performed with Cos-1 cells transfected with different plasmid combinations as indicated in the figure. DAPI was used to stain the nucleus (*blue* color). Merge I shows the merged images of RFP & GFP panel, while merge II shows the merged images of RFP, GFP, and DAPI. The bar is equivalent to 10 μm. *D*, TIP60 CD domain is required for TIP60-PXR chromatin binding. Cos-1 cells were transfected with indicated plasmids alone or in combinations, and subcellular fractionation was performed to prepare soluble and chromatin bound fractions. Western blot analysis was performed with isolated fractions to detect the presence of TIP60 & PXR (WT or their mutant forms). GAPDH and H4 were used as markers for soluble and chromatin bound fractions, respectively. *E*, fractionation experiments were performed similar to (D) to detect & confirm the chromatin binding of endogenous TIP60 & PXR proteins in HepG2 cells. Western blot image shows the presence of TIP60 & PXR proteins in different fractions. *F*, TIP60 regulates PXR chromatin binding. For transient knockdown of TIP60, HepG2 cells were transfected with siTIP60 or siGL2 (control) and after 24 h, subcellular fractions (soluble & chromatin bound) were prepared. Western blot analysis was performed with indicated antibodies to detect the presence of TIP60 & PXR proteins in isolated fractions. DBD, DNA-binding domain.

The expression of a gene depends on its local chromatin environment, which is regulated by epigenetic regulators and transcription factors. In this study, we have shown significant alteration in the transcriptional status of wound healing–related genes in the presence of TIP60-PXR. As we know, TIP60 is a well-recognized chromatin modifier protein and PXR is a transcription factor, therefore, we wanted to dissect the mechanism by which this complex could modulate the expression of targeted genes during wound healing. Interestingly, both TIP60 and PXR possess chromatin-binding abilities due to the presence of chromodomain (CD) and DNA-binding domain (DBD), respectively. Hence, we were interested to determine how these partners contribute to the loading of TIP60–PXR complex on to the chromatin. To examine this, we generated TIP60 CD mutant and PXR DBD–deleted mutant. Two highly conserved and essential amino acids (F43A, Y47A) present in the CD of TIP60 were mutated to generate TIP60 CD mutant, based on previously published studies (26, 27), while a DBD-deleted construct of PXR was generated by deleting 39 to 130 amino acids (24) of the DBD of full-length PXR (Fig. 4*A*) and subsequently cloned TIP60 (CD mutant) and PXR (ΔDBD) construct in RFP-tagged and GFP-tagged vectors, respectively (data not shown).

Before probing the effect of TIP60 CD mutation and PXR DBD deletion on loading of TIP60–PXR complex on chromatin, we examined the expression level and subcellular localization of these two constructs, by Western blotting and live cell imaging experiments, in transiently transfected Cos-1 cells with TIP60 (CD mutant) and PXR (ΔDBD) constructs alone or in combination. Western blot analysis results showed the expression of both RFP-TIP60 (CD mutant) and GFP-PXR (ΔDBD) constructs at their expected size (Fig. 4*B*). Interestingly, live cell imaging of Cos-1 cells expressing RFP-TIP60 (CD mutant) alone showed altered localization pattern of TIP60 (CD mutant) when compared with TIP60 (WT), as TIP60 (CD mutant) was observed to be homogenously distributed inside the nucleus in contrast to the punctate nuclear foci formed by TIP60 (WT) (Fig. 4*C*). Similarly, PXR (ΔDBD) predominantly localized in the cytosol, in clear contrast to the PXR (WT) that showed exclusive nuclear localization. Most importantly, we observed that TIP60 (CD mutant) failed to induce nuclear foci formation of both WT and DBD-deleted construct of PXR, while TIP60 (WT) could significantly translocate and formed colocalized foci with PXR (ΔDBD) inside the nucleus (Fig. 4*C*).

To analyze the interaction of TIP60 and PXR with chromatin, we performed subcellular fractionation of Cos-1 cells (transfected with different combinations of plasmids as indicated in Fig. 4*D*) using a gradient of salt concentrations, to separate the chromatin-bound fraction from the remaining cellular fraction consisting of cytoplasmic and nucleoplasmic fraction. In order to examine and verify the quality of these fractions, we performed Western blot analysis with GAPDH and histone H4 antibodies which showed the expected presence of cytoplasmic protein GAPDH in soluble fraction, while the presence of histone H4 was detected only in the chromatin fraction (Fig. 4*D*). Next, to monitor the presence of TIP60 and PXR in the two isolated fractions, Western blot analysis was performed and the results showed exclusive presence of TIP60 (WT) in the chromatin fraction, however, PXR (WT) was present predominantly in the soluble fraction (Fig. 4*D*). Interestingly, the introduction of point mutations in the TIP60 CD hindered its chromatin-binding ability as evident by its presence detected majorly in the soluble fraction (Fig. 4*D*). Under similar conditions, DBD-deletion construct of PXR was majorly detected in the soluble fraction.

Next, to examine the effect of these mutations on TIP60–PXR complex loading on to chromatin, subcellular fractionation was performed followed by Western blotting experiments with Cos-1 cells cotransfected with different combinations of plasmid as indicated in Figure 4*D*. Results showed that the presence of TIP60 (WT) induced significant shift of both PXR (WT) or PXR (ΔDBD) into chromatin fraction from the soluble fraction, however, TIP60 (CD mutant) was unable to bring about any such change in any of the two forms of PXR (WT or ΔDBD), and both remained present majorly in the soluble fraction (Fig. 4*D*). To further validate, we performed similar experiment with untransfected HepG2 cells to examine the chromatin-binding status of endogenous TIP60 and PXR. Western blot analysis of the chromatin bound and soluble fractions showed the complete presence of TIP60 and PXR protein only in the chromatin-bound fraction (Fig. 4*E*). To examine the effect of TIP60 on PXR chromatin binding, we performed siRNA-mediated knockdown of TIP60 in HepG2 cells, and soluble and chromatin bound fractions were prepared. Western blot analysis showed drastic reduction in the chromatin binding of PXR protein in the absence of TIP60 (Fig. 4*F*). Overall, the findings of these experiments demonstrate that TIP60 essentially mediate the chromatin loading of the TIP60–PXR complex *via* its CD.

**Figure 5:**
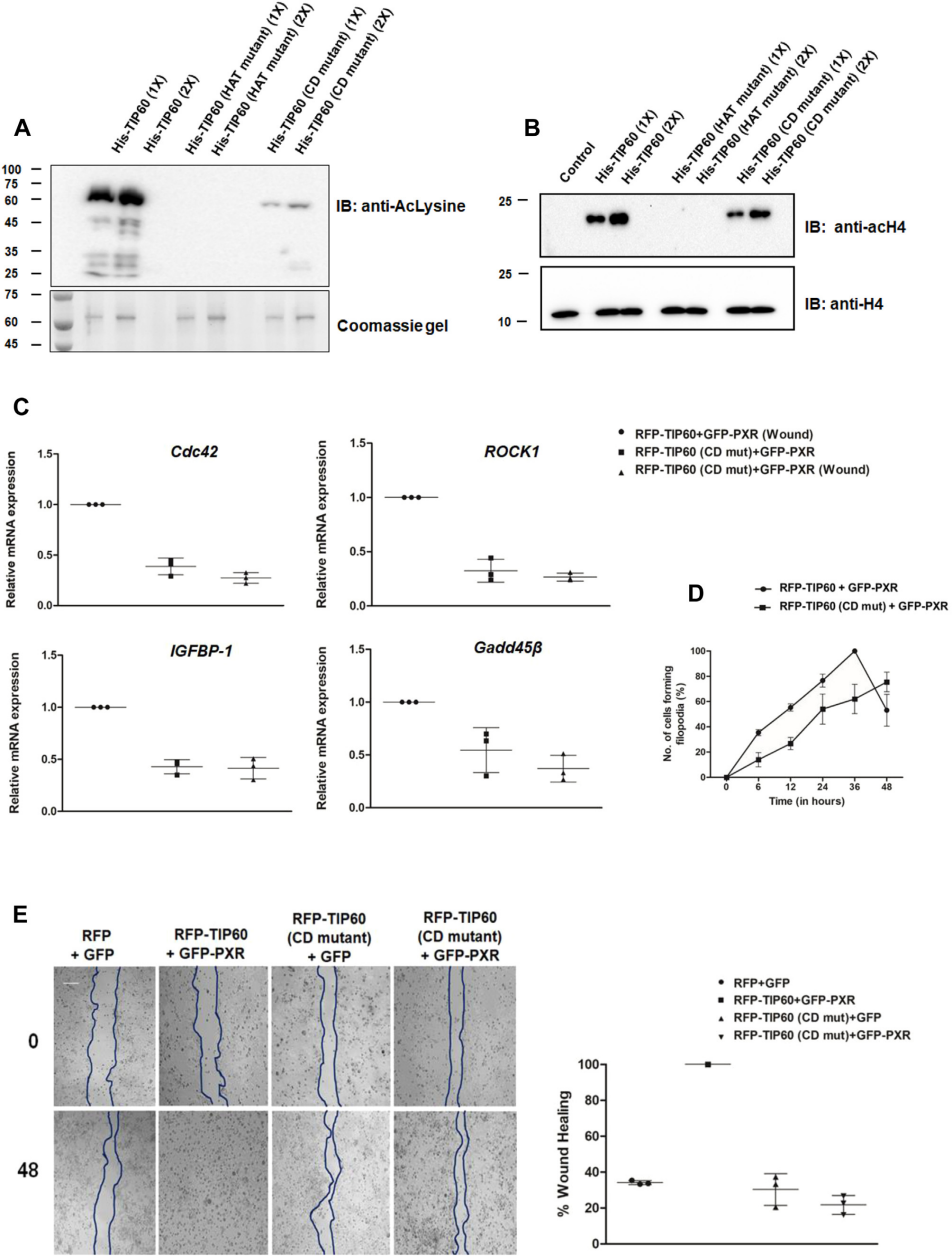
**TIP60 CD mutant exhibit diminished histone acetylation and wound healing capacity.***A*, mutation in TIP60 chromodomain compromise its autoacetylation activity *in vitro*. *In vitro* autoacetylation assay was performed using His-tagged TIP60 (WT), TIP60 (HAT mutant), and TIP60 (CD mutant) followed by Western blot analysis using anti-acetylated lysine antibody. Coomassie gel image shows the loading of recombinant proteins as indicated. *B*, TIP60 (CD mutant) exhibit diminished HAT activity. *In vitro* HAT assay was performed taking recombinant histone H4 protein as a substrate and TIP60 (WT) or its mutant forms as indicated. Western blot analysis was performed using anti-acetylated H4 antibody. Western blot with H4 antibody shows the equal loading of the samples. *C*, TIP60 (CD mutant) failed to activate TIP60-PXR targeted wound-related gene expression. RT-qPCR analysis was performed to analyze the expression of TIP60-PXR targeted wound-related genes in HepG2 cells expressing TIP60 (CD mutant) with PXR during control and wound-generated conditions. Scatter plots depict the average value of relative expression of genes for three independent experiments, taking value of TIP60 (WT) and PXR coexpressing cells during wound generated condition as 1. *p* values for (RFP-TIP60+GFP-PXR (Wound) *versus* RFP-TIP60 (CD mutant)+GFP-PXR or RFP-TIP60 (CD mutant)+GFP-PXR (Wound)) *Cdc42, ROCK1, IGFBP-1* is <0.0001 and for *GADD45β* is 0.0043. *D*, TIP60 (CD mutant) affects filopodia formation in HepG2 cells. HepG2 cells were transfected with indicated plasmids, and *in vitro* scratch was generated after 24 h of medium change. The edges of wound were monitored for 0, 6, 12, 24, 36, 48 h and graph was plotted for average number of cells (%) forming filopodia from three independent experimental replicates (±+S.D.). *p* values for 6, 12, and 24 h are 0.0037, 0.0009, and 0.0395, respectively. *E*, TIP60 (CD mutant) failed to fill the wound gap. Scratch was generated in HepG2 cells coexpressing indicated combinations of plasmids. Wound gap filling was monitored at 0 and 48 h post wound induction. Scatter plot show the average mean value of wound gap filling (%) at 48 h for three independent experimental replicates. *p* values (for RFP-TIP60+GFP-PXR versus RFP+GFP or RFP-TIP60 (CD mutant)+GFP or RFP-TIP60 (CD mutant)+GFP-PXR) is <0.0001. The bar is equivalent to 100 μm. CD, chromodomain.

Since our previous results showed that TIP60 acetylate histone H2B and H4 during wound healing and its HAT activity is important for its wound repair function, we wanted to determine the role of CD mutation (that renders TIP60 incapable of loading onto the chromatin) on its autoacetylation and HAT activity. *In vitro* autoacetylation assay performed with purified recombinant His-tagged TIP60 (WT), TIP60 (HAT mutant), and TIP60 (CD mutant) proteins showed drastic decrease in autoacetylation capacity of TIP60 (CD mutant) protein (Fig. 5*A*). Similarly, *in vitro* HAT assay was performed taking histone H4 as substrate and the result displayed reduced HAT activity of TIP60 (CD mutant) protein in comparison to TIP60 (WT) protein (Fig. 5*B*).

Based on our observations that TIP60 CD is crucial for formation and loading of TIP60–PXR complex onto the chromatin as well as its catalytic activity, we next wanted to examine the impact of TIP60–PXR complex chromatin loading inability on targeted gene expression and wound healing process. Real-time PCR analysis was performed with harvested HepG2 cell extracts expressing TIP60 (WT) or TIP60 (CD mutant) in combination with PXR during wound-generated conditions. The result showed that TIP60 (CD mutant) complex with PXR failed to induce the expression of candidate genes during wound-generated condition (Fig. 5*C*). Since TIP60 CD mutation impacted the wound-healing–induced gene activation, we also wanted to examine the effect of CD mutation on wound-induced filopodia formation in these cells. The result showed that in TIP60 CD mutant–expressing cells, there was a significant decrease in the number of cells forming filopodia (Fig. 5*D*).

Further, we examined the effect of CD mutation on TIP60 wound healing capability and performed *in vitro* wound-healing assay with transfected HepG2 cells (TIP60 (WT) or its CD mutant with PXR) and monitored the filling of wound gap at regular intervals up to 48 h. The result showed failure of wound gap closure in cells expressing TIP60 (CD mutant) and PXR even at 48 h, while under similar conditions, complete filling of wound gap was observed in the cells expressing TIP60 (WT) with PXR (Fig. 5*E*). Overall, these findings suggest that TIP60 CD-dependent TIP60–PXR complex chromatin loading is vital for the expression of genes regulating filopodia formation and cell migration required for wound closure.

## Discussion

So far, TIP60 has been extensively studied in context of its role in DNA repair process, while, PXR, a class II nuclear receptor is widely known for its role in regulating the expression of drug metabolizing enzymes and transporters (25). In this study, we have first time identified the role of TIP60-PXR as new players participating in the wound healing process *via* altering the actin dynamics and cellular motility of the cell. To support our proposition, we have provided evidence that TIP60-PXR enhance cell migration, in response to wound healing *via* initiating actin-reorganization and inducing early filopodia formation. Filopodia are thin actin-rich finger like extensions, filled with tight parallel bundles of filamentous (F)-actin and are commonly formed at the leading edge of migrating cells and play role in different cellular processes like wound healing, cell-cell adhesion, embryonic development, neuronal growth cone guidance, phagocytosis, and in sensing of cellular microenvironment (28, 29, 30). Our data clearly showed the change in cell morphology, with formation of filopodia extensions as early as 6 h of wounding in significant number of cells expressing TIP60 and PXR in response to wound stimuli that correlated with enhanced cellular motility (Fig. 1). On the contrary, filopodia formation dramatically reduced with the inhibition of TIP60’s catalytic activity which eventually affected the rate of cell migration and wound closure.

On screening of candidate genes modulated by TIP60/PXR in response to wounding, we found instantaneous enhancement in the expression of *Cdc42* and *ROCK1* genes that showed correlation with early formation of filopodia and enhanced migration of cells, expressing TIP60/PXR (Fig. 2*A*). The assembly of dynamic actin structures within the cells required for migration and establishment of polarity is mainly controlled by the coordinated actions of Cdc42, RhoA, and Rac1 (the three most extensively characterized members of the Rho family of small GTPases) and their interaction with various cellular proteins (31). Cdc42 plays a vital role in inducing the formation of filopodia extensions and maintaining cell polarity toward the leading edge during cell migration, while RhoA triggers the assembly of stress fibers by inducing the action of downstream effectors, including ROCK1 (Rho-associated coiled coil-containing protein kinase) and confer contractility to the cells undergoing locomotion (29, 32, 33, 34, 35). ROCK1 has been shown to play a role in stabilizing actin filament by inhibiting their depolymerization and inducing front-back polarity in migrating cells (36). Correspondingly, there are reports which show that inhibition of ROCK1 activity can inhibit cell migration in a variety of cells (37, 38, 39). In addition, we have detected postwounding enhancement in the expression of *Gadd45β* and *IGFBP-1* genes in cells coexpressing TIP60 and PXR (Fig. 2*A*). IGFBP-1 (insulin-like growth factor binding protein) has been shown to accelerate cutaneous wound healing in mice and rabbit models and induce cell migration properties of extravillous trophoblast cells, human dermal fibroblast cells, and HepG2 cells (40, 41, 42, 43, 44, 45). Chesik D et al. revealed the role of IGFBP-1 in increasing F-actin polymerization and in inducing migration of oligodendrocytes (46). Similarly, Gadd45β (the growth arrest and DNA damage-inducible 45 beta), an important regulator of stress signaling that acts in response to physiological and environmental stress, has been shown to modulate cell migration and epithelial-mesenchymal transition of HuCCA-1 cells (47, 48). Interestingly, both IGFBP-1 and Gadd45β has been identified as PXR-regulated factors, where PXR-mediated differential activation of IGFBP-1 and Gadd45β signaling pathways has been shown to stimulate morphological changes to favor migration of ShP51 cells (49). PXR is reported to bind to the promoter of *Gadd45β* gene to stimulate its mRNA expression that results in actin filament reorganization and cell migration (49). Similarly, PXR has been shown to activate *IGFBP-1* by repressing *HNF4α* gene leading to altered cell morphology and migration (45). In accordance with these findings, our ChIP-qPCR assay result demonstrated wound-induced chromatin binding of TIP60 on one of the screened gene promoter, *ROCK1* (Fig. 2*C*) and suggested that TIP60–PXR complex acts as a molecular switch to activate the downstream signaling pathways thereby controlling actin reorganization, filopodia formation, and cell migration during wound healing. Interestingly, we observed time-dependent, differential activation pattern of the screened TIP60-PXR targeted genes in response to wounding. While *Cdc42, ROCK1*, and *Gadd45β* showed early activation right after wound induction, the enhancement in *IGFBP-1* expression was detected at 6 h post wound induction (Fig. 2*B*). This suggest that some gene promoters might be occupied early by TIP60–PXR complex for inducing immediate repair action against injury, while other targeted gene promoters are occupied subsequently to prepare cell for inducing holistic response. It would be interesting to identify the global changes in gene expression induced by TIP60-PXR to facilitate wound repair process and examine the mechanism regulating the context-dependent activity of TIP60.

We know that epigenetic landscape never remains static and is always changing to respond to the changes in cellular microenvironment. Epigenetic modifications and chromatin reorganization play an important role in determining the expression profile of genes and TIP60 being a well-documented HAT protein, provided the rationale to explore and define the role of TIP60 in inducing epigenetic changes, which may potentially regulate or fine-tune the temporal gene expression occurring during wound healing process.

TIP60 has been shown to acetylate H2A, H3, and H4 and its CD facilitates its interaction with various histones (50, 51, 52). In contrast to these findings, when we examined the histone-binding preference of TIP60 under wound-generated condition, we observed that along with H4, TIP60 also acetylated H2B upon wound induction. This exemplifies that TIP60 can alter its chromatin modification preferences according to prevailing cellular environment. Interestingly, a study has shown that stress to embryonic stem cells enhance histone H2B acetylation and leads to cytoskeletal reorganization through CFL2/F-actin dynamics (53). Core histones (H2B/H4) are also shown to be hyperacetylated during spatial memory formation in dorsal hippocampus of rat (54). Our findings corroborate with other studies which showed enhanced acetylation of histone H4 post-wound induction. According to latest research findings by Nascimento-Filho CHV *et al.,* it has been shown that histone modification pattern alters during different stages of healing and may also vary depending on the state and location of the surrounding cells from the injured area (55). Further they observed that, migrating epithelial cells showed remarkably enhanced H4K12 hyperacetylation in early stages of wound healing, while histone H4K16 was the only lysine found to be hyperacetylated in the epithelial cells of closed wound areas. As the healing progressed, continuous deacetylation was noted at other lysine sites of histone H4 (H4K5, H4K8, and H4K16) (55). Similarly, studies using phototherapy for skin and mucosal wound treatment have shown to augment acetylation levels of histone H3 (H3K9ac) and lead to enhanced cell migration and wound healing (56, 57). There are numerous evidences which show that compounds inhibiting HDAC activity promote wound healing and regeneration in different kind of tissue injuries (58, 59, 60, 61, 62). For instance, treatment of HDAC inhibitors such as Tricostatin A alone or in combination with DNA methyltransferase inhibitor (5-aza-dC) increase the regeneration of amputated digit in mice (58). Similarly, valproic acid (VPA) (a HDAC inhibitor) showed improved recovery of damaged nerves in spinal cord injury (SCI) animal models (59, 60). Valproic acid is shown to inhibit HDAC and prevent reduction in histone H3 and H4 acetylation levels in the injured spinal cord of the rat model of SCI. Another study showed that HDAC6 negatively regulates wound healing in diabetic mice, and interestingly Tubastatin A (a HDAC6 inhibitor) treatment accelerates wound healing in diabetic mice, and nocodozole (acetylation inhibitor) can negate tubastatin effect (62). All these findings reveal the importance of acetyl transferases and histone acetylation in regulating gene expression during wound healing. Thus, we speculate that TIP60-PXR induced histone modifications at the promoters of the targeted genes prime them for active transcription during wound healing.

**Figure 6:**
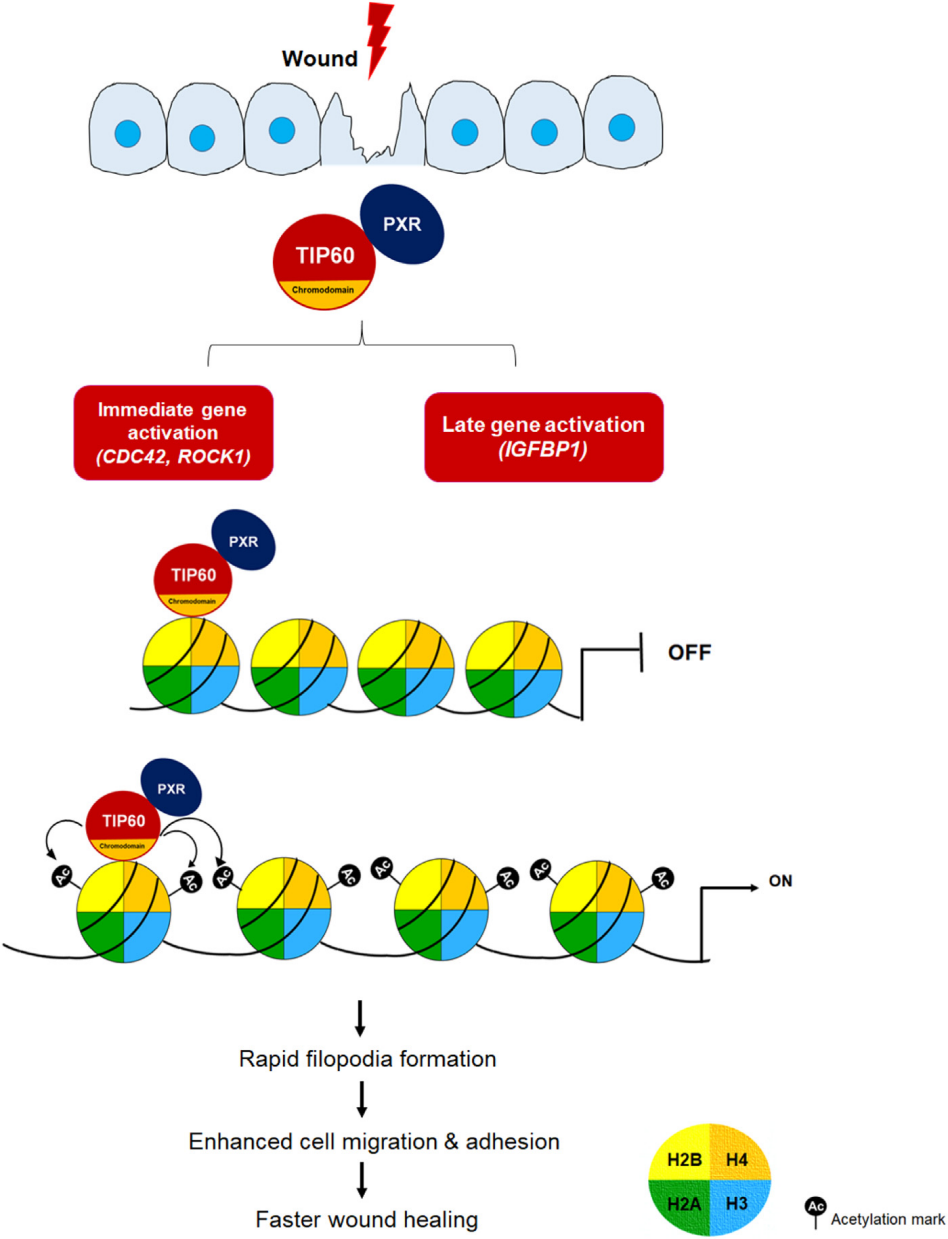
**Schematic diagram showing the role of TIP60–PXR complex in wound repair phenomenon.** Upon wound induction, TIP60–PXR complex, that is either already present on the promoter of its target genes or bind on to promoter through TIP60’s chromodomain, leads to enhanced acetylation of histone H2B and H4. These epigenetic modifications of local chromatin environment trigger immediate activation of *Cdc42*, *ROCK1*, *GADD45β*, and late activation of *IGFBP-1* gene post wound induction. Upregulation of these genes leads to actin reorganization & early filopodia formation promoting migratory capability of cells that subsequently leads to faster wound healing.

We identified that loading of TIP60–PXR complex onto the chromatin is dependent on TIP60. Mutating TIP60’s CD hampered TIP60–PXR complex chromatin binding, as well as TIP60’s autoacetylation and catalytic activity, critically required for inducing histone acetylation and priming targeted genes for transcription. This eventually affected the filopodia formation, cell migration, and wound-repair ability of cells. In a previous study from our lab, we have shown that PXR can augment TIP60 catalytic HAT activity (24). These findings suggest that enhanced lysine acetyl transferase activity of TIP60–PXR complex induce hyperacetylation of targeted histones during wound repair. Despite limited repair and regeneration capacity in humans, the liver is an organ with remarkable potential for repair and regeneration and is notably one of the few organs in which PXR is highly expressed (63). In context of these facts and our findings, there seems to be a probable relation between healing and regeneration capacity of organs, and hence expression level of TIP60 and PXR in them would be interesting to examine experimentally. In conclusion, our study shows that during mechanical injury to the cells, TIP60–PXR complex acetylate H2B and H4 at targeted gene promoters leading to active transcription of genes involved in actin remodeling and filopodia formation, resulting in enhanced cell migration and rapid wound closure (Fig. 6).

## Experimental procedures

### Reagents

Dulbecco’s Modified Eagle Medium (DMEM) (Gibco), fetal bovine serum (Gibco), penicillin-streptomycin (pen/strep) (Gibco), trypsin-EDTA (Gibco), histone antibodies (Cell Signalling Technology), GAPDH antibody (Abgenex), TIP60 antibody (Santa Cruz Biotechnology), PXR antibody (Santa Cruz Biotechnology), β-actin antibody (Santa Cruz Biotechnology), lipofectamine 2000 (Invitrogen), lipofectamine RNAiMAX reagent (Invitrogen), Wizard SV gel and PCR clean up system (Promega), Ni-nitrilotriacetic acid (Ni-NTA) (Genetix), histone 4 peptide (Millipore), Alexa Fluor 594 phalloidin (Invitrogen), NU9056 (Tocris), Verso cDNA synthesis kit (Thermo Scientific), SYBR green (Applied Biosystems), TRIzol reagent (Life technologies), DAPI antifade mountant (Invitrogen), horseradish peroxidase–conjugated heavy chain secondary mice and rabbit antibodies (Santa Cruz Biotechnology), light chain–specific horseradish peroxidase–conjugated secondary mice antibody (Merck), and Protein A-Sepharose 4B conjugate bead (Invitrogen). Primers used in the study were synthesized from Integrated DNA technologies and Eurofins Genomics. Primer sequences are mentioned in supporting information (Table S2). siRNA for TIP60 was purchased from Santa Cruz Biotechnology, while siGL2 (used as control) was synthesized from Sigma, with target sequence ‘CGTACGCGGAATACTTCGA’ (24).

### Cell culture, transfections, and live cell imaging

Cos-1 (monkey kidney fibroblast-like cell line), HepG2, and Huh-7 (human hepatocarcinoma-derived cell lines) cells were obtained from Cell Repository, NCCS, Pune, India. Cells were cultured in DMEM containing 10% FBS and 0.5% pen/strep solution. Cells were maintained at 37 °C, supplied with 5% CO_2_ under humid conditions, and subconfluent cells were passaged every third day using 0.5% Trypsin-EDTA. To determine the localization of fluorescent-tagged proteins, live cell imaging was performed in transiently transfected cells. For transient transfection, cells were seeded with 70% confluency and allowed to attach for 8 h followed by transfection with plasmids using lipofectamine 2000 reagent following manufacturer’s protocol. Posttransfection, medium was replaced with DMEM supplemented with charcoal-stripped FBS and after 24 h of medium change, cells were monitored under Nikon Ti Eclipse fluorescence microscope (Nikon) to visualize the localization of fluorescent-tagged proteins. For performing siRNA-based TIP60 knockdown, cells were transfected with 30 nM of siTIP60 or siGL2 (control) duplex using lipofectamine RNAiMAX reagent as per manufacturer’s protocol. Twenty-four hours post medium change, cells were harvested for Western blot analysis.

### Plasmid constructs and recombinant protein purification

To generate TIP60 CD mutant, two residues, phenylalanine at position 43 and tyrosine at position 47 were converted into alanine by overlapping PCR method (26, 27). To amplify TIP60 (CD mutant) ORF, PCR-1 reaction was performed using hTIP60-KpnI-Fw (forward), hTIP60-Chromodomain double mutant-Rv (reverse) primers, while PCR-2 reaction was performed using hTIP60-Chromodomain double mutant Fw and hTIP60-BamHI-Rv primers and taking TIP60 (WT) ORF as a template. To amplify TIP60 (CD mutant) ORF, PCR-3 was performed using PCR-1 and PCR-2 products as the template and hTIP60-KpnI-Fw and hTIP60-BamHI-Rv primer sets. The amplified product was further purified using Wizard SV gel and PCR clean up system. The purified amplicon was digested using KpnI and BamHI enzymes and subsequently cloned into digested pDsRed vector. To clone TIP60 (CD mutant) into pET28a vector, full-length TIP60 (CD mutant) ORF was amplified using hTIP60-BamHI-Fw and hTIP60-EcoRI-Rv primers set and taking RFP-TIP60 (CD mutant) clone as the template. Amplified PCR product and pET28a vector were digested using BamHI and EcoRI enzymes, followed by ligation to generate pET28a-TIP60 (CD mutant) construct. Similarly, DBD-deleted PXR amplicon was amplified using hPXRNterm-EcoRI Fw and hPXR-DBD deletion mutant Rv and hPXR-DBD deletion mutant Fw and hPXR LBD-BamHI Rv primer sets by overlapping PCR method. The amplified product was digested with EcoRI and BamHI enzymes and subsequently cloned into pEGFP vector. His-TIP60 (WT) and His-TIP60 (HAT mutant) (mutated at Q377E, G380E) constructs were previously cloned in the lab (24).

For recombinant protein expression, BL21 DE3 codon plus cells were transformed with pET28a-TIP60 (WT), pET28a-TIP60 (CD mutant), or pET28a-TIP60 (HAT mutant) plasmids, and transformed cells were grown in Luria Bertani broth at 37 °C until the optical density of culture reached 0.6. Subsequently, 0.5 mM of IPTG was added in the culture and incubated for 16 h at 16 °C to induce recombinant protein expression. Post induction, bacterial cells were pelleted at 3000*g* for 10 min at 4 °C, and obtained bacterial cell pellet was lysed in lysis buffer (1X PBS, 2 mM EDTA, 5 mM DTT, 0.5 mM PMSF, 0.1% Triton X 100, 10% glycerol, and 100 μg lysozyme). Lysate was sonicated for three cycles at 30% amplitude and incubated for 1 h at 4 °C with continuous rotation followed by centrifugation at 14,000*g* for 30 min to obtain clear supernatant. Ni-NTA beads were washed twice with 1X PBS and once in lysis buffer. Equilibrated Ni-NTA beads were then added to supernatant and rotated at slow speed for 1 h at 4 °C. Supernatant was then centrifuged at 300*g* for 5 min, and beads were washed twice with wash buffer (1X PBS, 0.1 mM PMSF, 20 mM imidazole). Bead-bound proteins were then eluted in elution buffer (50 mM Tris-pH 8.0, 10 % glycerol, 150 mM NaCl, 500 mM imidazole). Eluted proteins were dialyzed in HAT buffer (50 mM Tris-pH 8.0, 10% glycerol, 0.1 mM, 1 mM DTT, 20 μM PMSF) for 4 h and these dialyzed proteins were further used for performing *in vitro* assays.

### Subcellular fractionation, Western blot analysis, and immunoprecipitation assay

For preparing soluble fraction (cytoplasmic and soluble nuclear fraction) and chromatin-bound fraction, Cos-1 cells were seeded at 70% confluency and transfected using respective plasmids. For endogenous subcellular fractionation, HepG2 cells were seeded at 70% confluency and siRNA-based TIP60 knockdown was performed as described previously. After 24 h of medium change, cells were harvested and washed twice with 1X PBS buffer and then lysed in soluble lysis buffer (10 mM Hepes pH-7.4, 10 mM KCl, 0.05% NP-40, 0.2 mM MgCl_2_, 1% Triton X 100, 100 mM NaCl, 1X protease inhibitor cocktail) for 20 min at 4 °C. Cell lysate was then centrifuged for 5 min at 1300*g* using benchtop cold centrifuge, and supernatant (soluble fraction) was gently aspirated without disturbing the pellet. Remaining pellet was then washed twice with soluble lysis buffer and resuspended in chromatin lysis buffer (50 mM Tris pH-8.0, 400 mM NaCl, 10 mM EDTA, 0.5% SDS, 1X protease inhibitor cocktail) following incubation at 4 °C for 20 min. Subsequently, the lysate was sonicated twice at 20% amplitude for 20 s on and 30 s off cycle followed by centrifugation at 1700*g* for 5 min at 4 °C, and supernatant was collected as chromatin fraction. Further, protein quantification was performed for both the fractions using Bradford reagent, and 10 μg/μl of samples were boiled with 2X Laemmli sample buffer followed by Western blotting.

For Western blot analysis, protein samples were boiled with 2X Laemmli sample buffer and then resolved in SDS-PAGE gel, followed by transfer on methanol-charged PVDF membrane using semi-dry transfer method. Protein-bound membrane was incubated with 5% skimmed milk for 1 h and washed with 1X PBS buffer followed by overnight incubation with primary antibody at 4 °C and subsequently with secondary antibody for 1 h at room temperature. ECL reagent was used to develop the signal using FluorchemM system (Protein Simple). Full-length images for Western blots are provided in Fig. S4.

For immunoprecipitation experiments, HepG2 cells (normal and wound-generated) and transfected Cos-1 cells were harvested and lysed in lysis buffer (20 mM Tris pH-8.0, 2 mM EDTA, 150 mM NaCl, 0.5% Triton X 100, 0.1% SDS, 1X protease inhibitor cocktail) at 4 °C for 1 h followed by centrifugation at 14,000*g* at 4 °C. Supernatant was collected in a fresh vial, and 10% of supernatant was separated as input sample. The lysate was precleared by incubating with equilibrated protein-A Sepharose beads for 1 h at 4 °C to inhibit nonspecific protein binding onto beads. TIP60 antibody was added into this precleared lysate followed by addition of equilibrated protein-A sepharose beads. After overnight incubation at 4 °C, beads were separated by centrifugation at 300*g* and washed twice with lysis buffer. Immunoprecipitated proteins were eluted by adding 2X Laemmli sample buffer into the beads and boiling them at 95 °C for 10 min. Samples were further resolved in SDS-PAGE and resolved proteins were transferred onto PVDF membrane. Membrane was further incubated first with specific primary antibody and later with secondary heavy chain or light chain antibody. Blot was developed using ECL reagent and FluorchemM system (Protein Simple).

### HAT assay and autoacetylation assay

For HAT assay, purified recombinant His-TIP60 (WT), His-TIP60 (CD mutant), and His-TIP60 (HAT mutant) were incubated with 100 μM acetyl Coenzyme A and 0.5 μg histone 4 peptide for 1 h at 30 °C. Reaction was stopped by adding 2X Laemmli sample buffer and boiling at 95 °C for 10 min. Samples were resolved in 15% SDS-PAGE gel followed by Western blot analysis using anti-acetylated H4 or anti-H4 antibody. Similarly, for autoacetylation assay, equal concentration of purified recombinant His-TIP60 (WT), His-TIP60 (CD mutant), and His-TIP60 (HAT mutant) were incubated with 100 μM acetyl Coenzyme A for 1 h at 30 °C, and reaction was stopped by adding 2X Laemmli sample buffer. Samples were boiled at 95 °C for 10 min and resolved on 10% SDS-PAGE. Western blot analysis was performed using anti-acetylated lysine antibody.

### *In vitro* wound healing assay, filopodia formation, and actin staining

For *in vitro* wound healing assay, HepG2 cells were seeded at 80% confluency on 35 mm plates and transfected with respective plasmids. After 24 h of medium change, a uniform scratch was generated using a sterile 10 μl pipette tip, in a completely confluent monolayer of transfected HepG2 cells. Simultaneously, cell medium was changed with serum-free medium and the filling of wound gap was examined microscopically using Nikon Ti Eclipse fluorescence microscope (Nikon) at specified time intervals. High resolution images were captured by a digital camera attached to the microscope and computer system. To calculate the wound gap filled (in %), distance between different wound areas were measured using measurement and annotation tool provided with Nikon Ti Eclipse software. Wound gap closure at different time points was calculated by applying following formula on the averaged measured values from different wound areas.

$$\%\;Wound\;gap\;=\frac{\left(Scratch\;wound\;at\;t_{0}\;-\;Scratch\;wound\;at\;t_{6,12,24,36,48}\right)\;x\;100}{Scratch\;wound\;at\;t_{0}}$$

To examine the formation of filopodia, HepG2 and Huh-7 cells were seeded on glass coverslips and transfected with indicated plasmids using lipofectamine reagent followed by wound generation. To examine the morphology of the cells for formation of filopodia near wound edge, cells on glass coverslips were washed thrice with 1X PBS and then fixed with 4% formaldehyde at room temperature. After 15 min, fixative was removed and cells were again washed with 1X PBS. Fixed cells were then permeabilized with 0.1% Triton X 100 for 5 min at room temperature and subsequently, cells were washed thrice with 1X PBS and blocked with 1% bovine serum albumin for 20 min. For actin staining, cells were washed twice with 1X PBS and then incubated with Alexa Fluor 594 Phalloidin for 30 min in humidified chamber. Coverslips were then rinsed thrice with 1X PBS containing 0.1 % tween 20 and mounted on glass slide using DAPI antifade mountant. Prepared slides were then proceeded for analysis using Nikon Ti2 Eclipse confocal microscope (Nikon). Time course analysis of number of cells forming filopodia at different time-points (0, 6, 12, 24, 36, and 48 h) post wound induction was performed by recording number of cells forming filopodia using Nikon Ti2 Eclipse confocal microscope. Similar experiments were performed to examine the effect of TIP60 knockdown by siRNA- and NU9056-mediated TIP60 catalytic activity inhibition on filopodia formation and wound gap closure.

### RT-qPCR analysis and ChIP-qPCR analysis

To measure the effect of TIP60/PXR on wound-repair related gene expression, total RNA was isolated from transfected HepG2 cells (control or wound-generated condition) using TRIzol reagent. Isolated RNA was further processed for DNase treatment for 10 min at 37 °C followed by complementary DNA (cDNA) synthesis using Verso cDNA synthesis kit, as per manufacturer’s protocol. Prior to cDNA synthesis, RNA quantification was performed and equal concentration of RNA was used for cDNA synthesis for all the samples. Further, qPCR was performed with synthesized cDNAs, gene specific primers (Table S2), SYBR green PCR master mix using StepOneplus Real-time PCR system (Applied Biosystems, USA). Ct values obtained were used for calculating relative mRNA expression using formula, relative quantification (RQ) = 2ˆ(-ΔΔCt). *GAPDH* was used as an endogenous control and gene expression was calculated after normalizing values for all the genes with *GAPDH*. Upregulation of *Cdc42* and *ROCK1* genes was also measured with other endogenous controls *β-actin* and *28S* rRNA (Fig. S2).

To perform ChIP-qPCR, *in vitro* wound was generated in transfected HepG2 cells, while unwounded cells were used as a control. Cells were cross-linked with 37% w/v formaldehyde after 24 h of wound generation and subsequently quenched using 2.5 M glycine. Cells were then lysed in lysis buffer (0.5% SDS, 10 mM EDTA, 50 mM Tris pH-8.0, 1X protease inhibitor cocktail) for 30 min followed by sonication. Further, preclearing of sonicated lysate was performed (as mentioned in immunoprecipitation assay), and TIP60 antibody and protein-A sepharose beads were added into the precleared samples, followed by overnight incubation at 4 °C. Beads were then collected by centrifugation and washed with wash buffer (0.1% SDS, 1% Triton X-100, 2.5 mM EDTA, 20 mM Tris pH-8.0, 150 mM NaCl) followed by elution using elution buffer (1% SDS, 10 mM NaHCO_3_). Eluted samples were treated with 200 mM NaCl and subjected for overnight crosslink reversal at 65 °C. Samples were then incubated with proteinase K, 10 mM EDTA, and Tris pH-6.8 for 1 h at 45 °C, followed by RNase treatment for 30 min at 37 °C. ChIP-DNA was then recovered using phenol-chloroform–mediated extraction. Further, DNA was precipitated by adding 2.5 volumes of ice-chilled ethanol for 4 h in cold condition. Precipitated DNA was recovered by centrifugation at 14,000*g* followed by washing with 70% ethanol. To examine the binding of TIP60 on *ROCK1* promoter, qPCR reactions were performed using isolated ChIP-DNA as template and *ROCK1* gene promoter specific primers (Table S2).

### Statistical significance analysis

Statistical significance between two samples was calculated using unpaired *t* test. Statistical comparison for multiple samples were performed using one-way ANOVA with Bonferroni’s multiple comparison analysis. Statistical significance was considered with *p* value; *p* ≤0.05 as significant in this study. All data analysis for statistical significance and graphs displaying mean values (±S.D.) were constructed using Graphpad prism 5 software.

## Data availability

All data generated or analyzed during this study are included in this article (and its supporting information file).

## Supporting information

This article contains supporting information.

## Conflict of interest

The authors declare that they have no conflicts of interest with the contents of this article.

## Supporting information

Supporting Information (REVISION-2).Doc
