# Supplementary material for: TIP60 acts as a regulator of genes involved in filopodia formation and cell migration during wound healing
Source: J Biol Chem. 2022 May 5;298(7):102015. doi: 10.1016/j.jbc.2022.102015 (PMC9249863; doi:10.1016/j.jbc.2022.102015)
Supplement: Supporting Information (REVISION-2).Doc [file mmc1.doc]

**Supporting Information**

**Title:** TIP60 acts as a regulator of genes involved in filopodia formation and cell migration during wound healing

**Authors:** Shraddha Dubey^1^, Bharti Jaiswal^2^ and Ashish Gupta^1#^

**Supporting Figure 1: TIP60-PXR complex promotes filopodia formation in Huh-7 cells. (A)** Huh-7 cells were transfected with different plasmid combinations and wound was generated in confluent monolayer of cells. Filopodia formed in the cells at wound edges were monitored at 0, 6, 12, 24, 36 and 48 hours post wound induction. Graph represents the mean average value of percentage of cells forming filopodia from two independent experiments performed in duplicates. **(B)** Huh-7 cells were transfected with mentioned plasmids and generated wound was monitored for gap closure at different time intervals as indicated. Mean value calculated with +S.D. for three independent experimental replicates. P values for 12, 24 and 36 hour time points are 0.0002, <0.0001 and <0.0001, respectively.

**Supporting Figure 2: *Cdc42* and *ROCK1* genes shows similar fold induction in expression by TIP60-PXR complex under wound-induced condition irrespective of endogenous controls. (A)** HepG2 cells were overexpressed with plasmid combinations as mentioned in the graph and after 24 hours of wound generation, RT-qPCR analysis was performed. Scatter plots depict the relative mRNA expression levels of *Cdc42* normalized with *GAPDH, β-actin* or *28S* rRNA respectively. Average values for three independent experimental replicates were calculated with + S.D. P values (for *Cdc42/GAPDH, Cdc42/β-actin* and *Cdc42/28S* rRNA) are 0.0081, 0.0033 and 0.0019, respectively. **(B)** RT-qPCR analysis of *ROCK1* gene was performed as mentioned in (A). Relative mRNA expression levels for *ROCK1* gene was calculated by normalizing the values of *ROCK1* with *GAPDH, β-actin* or *28S* rRNA respectively. Graph depicts average mean value of three independent experimental replicates with + S.D. P values (for *ROCK1/GAPDH, ROCK1/β-actin* and *ROCK1/28S* rRNA) are 0.0104, 0.0018 and 0.0015, respectively.

**Supporting Figure 3: siRNA-mediated knockdown of TIP60.** HepG2 cells were transiently transfected with siRNA duplex of siTIP60 or siGL2 (Control) followed by transfection of RFP-TIP60 plasmid. Protein samples were resolved in SDS-PAGE followed by Western blot analysis using TIP60 antibody. Western blot with GAPDH was performed as loading control.

**Supporting Figure 4:** Full length blots for Western blots used in manuscript. Raw images for Figure **3A, 3B, 3C, 4B, 4D, 4E, 4F, 5A** and **5B**.

**Supporting Figure 1**


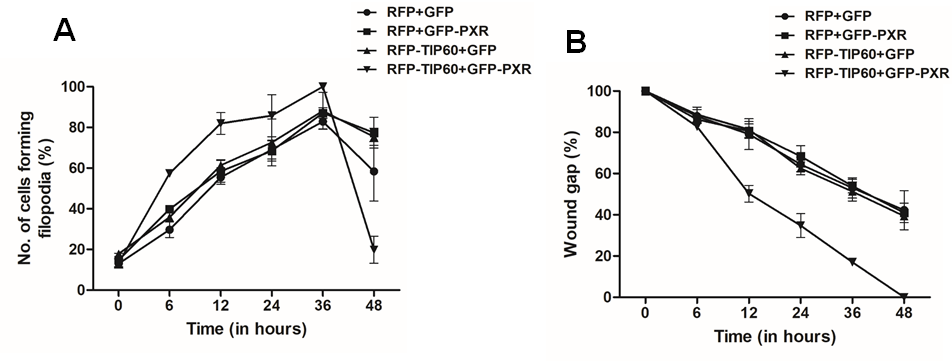


**Supporting Figure 2**


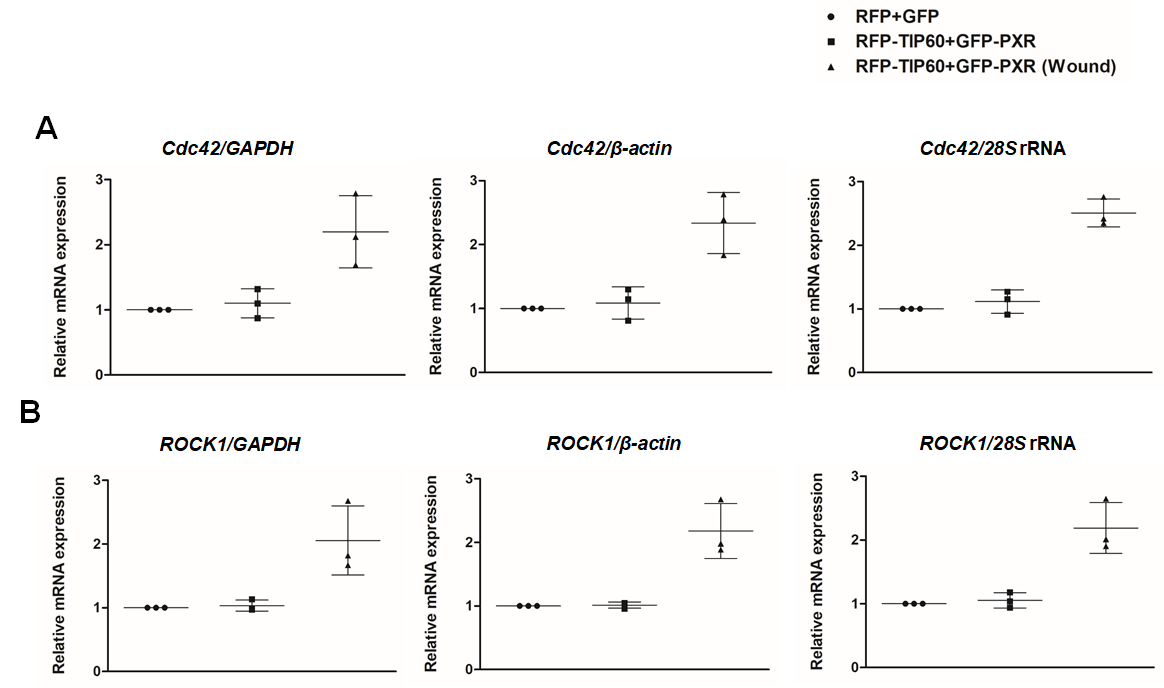


**Supporting Figure 3**


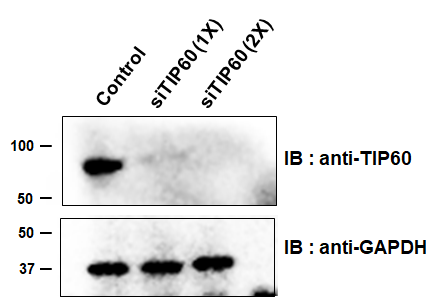


**Supporting Figure 4**

***(A) Raw images for Figure 3A***


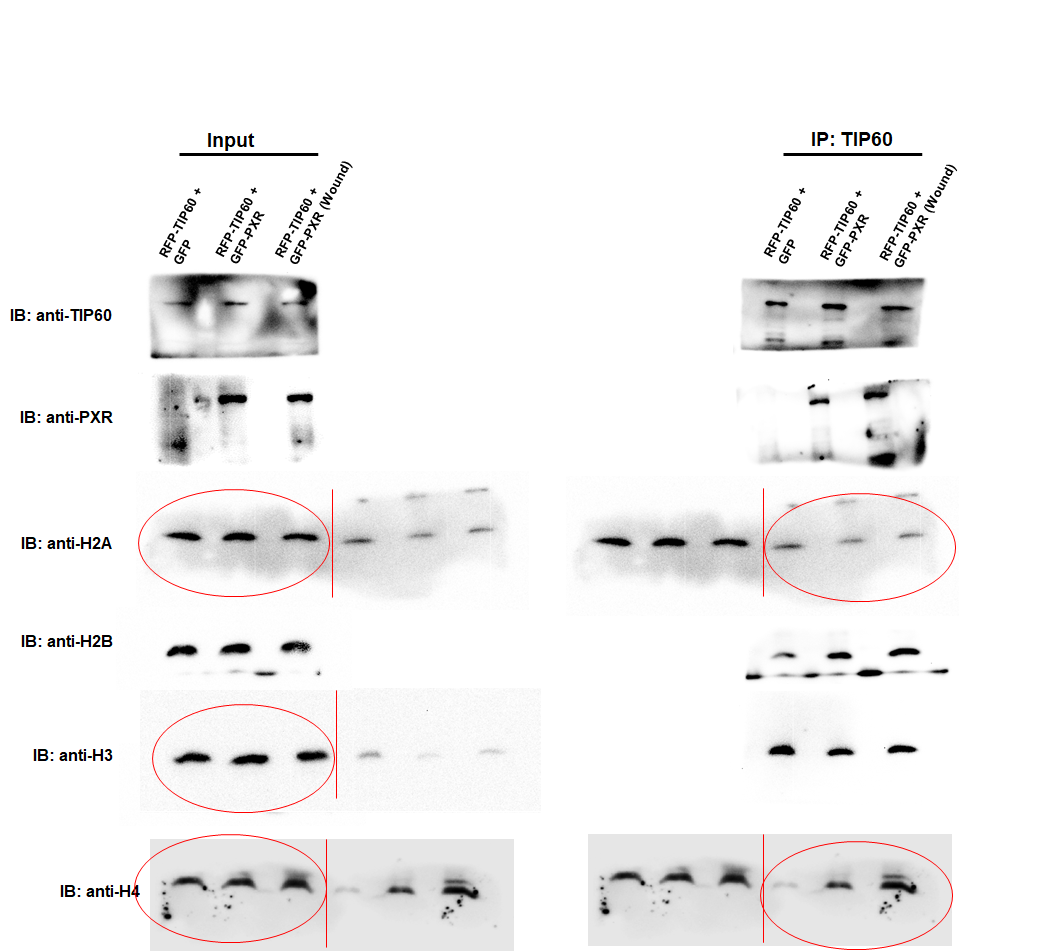


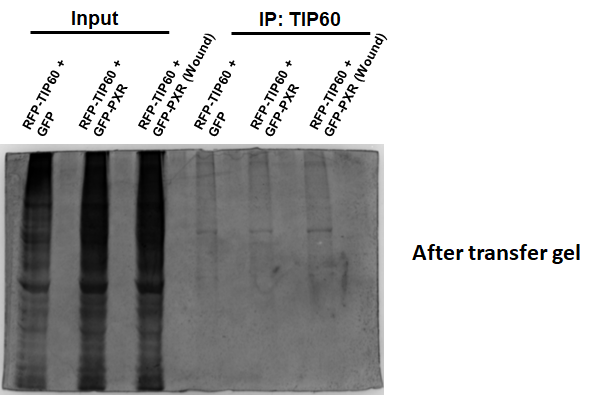


***(B) Raw images for Figure 3B***


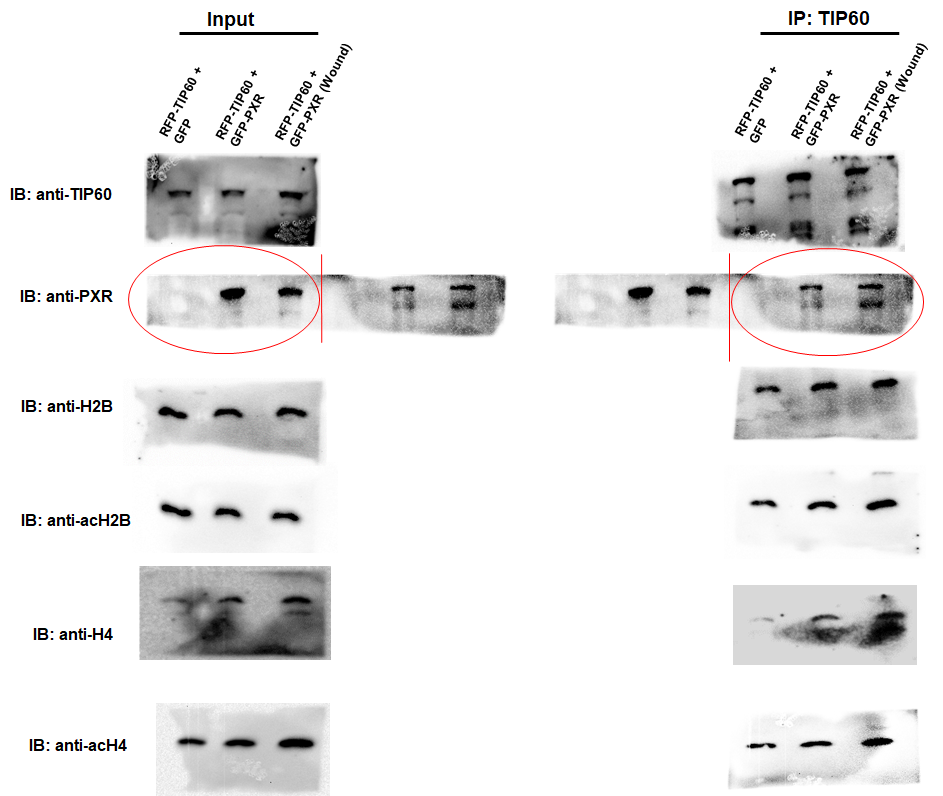


***(C) Raw images for Figure 3C***


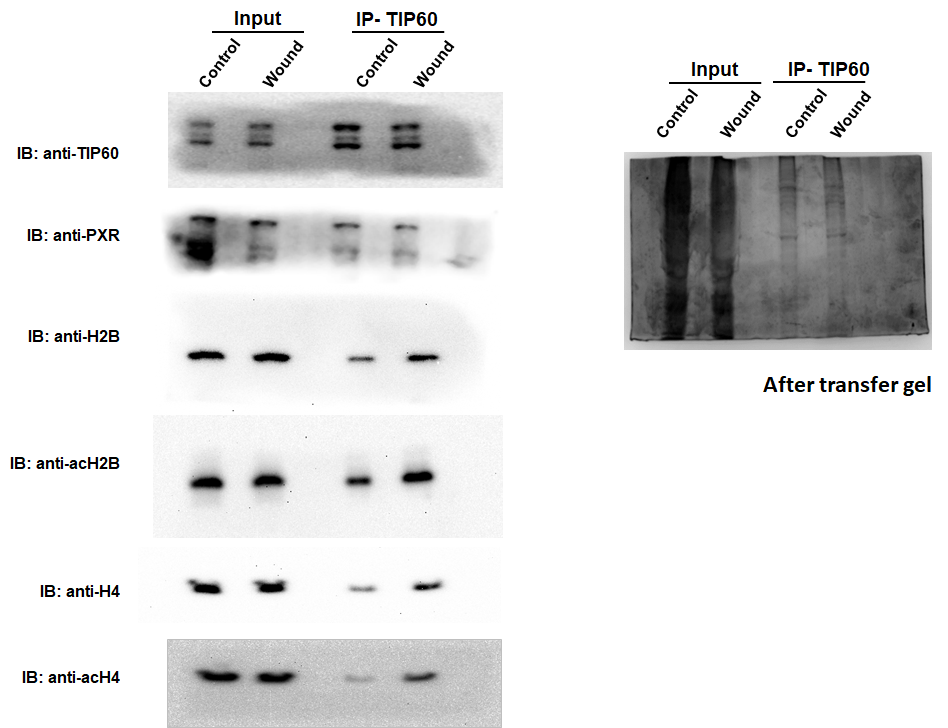


***(D) Raw images for Figure 4B***


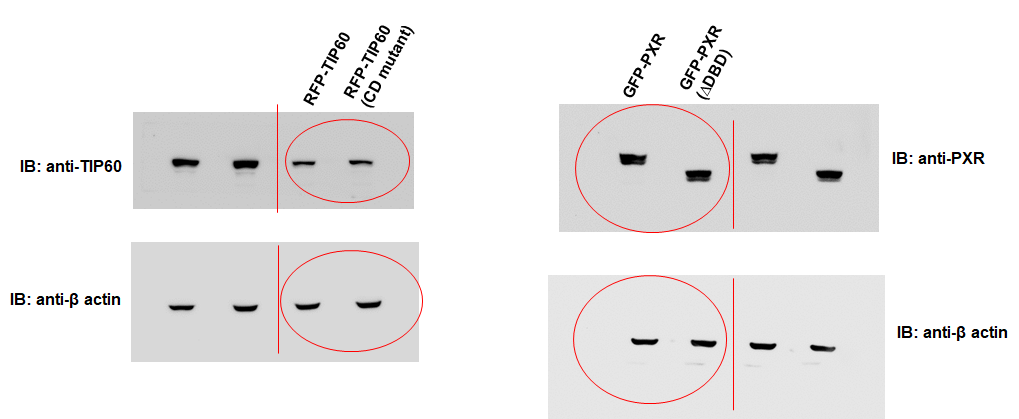


***(E) Raw images for Figure 4D***


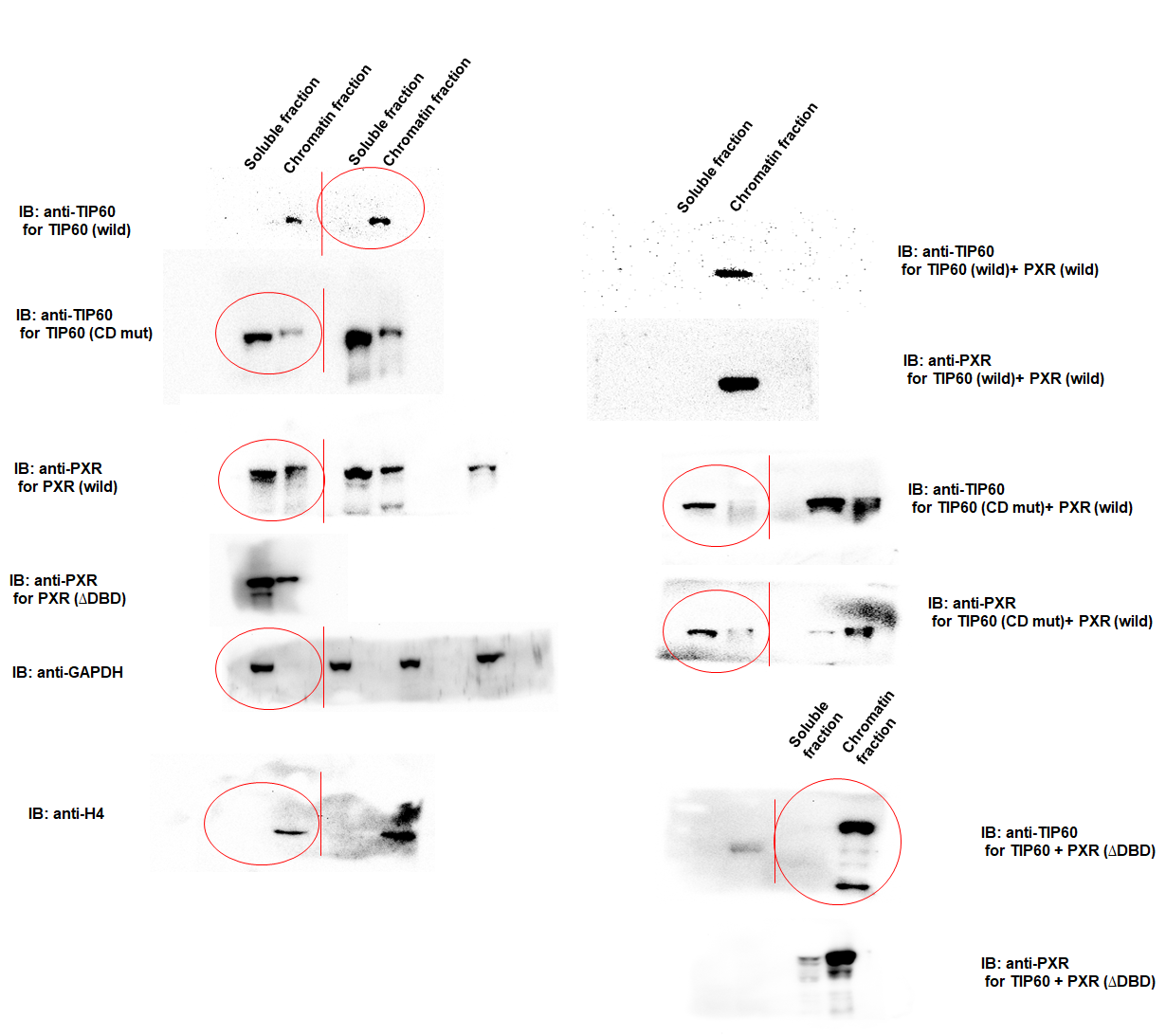


***(F) Raw images for Figure 4E & 4F***


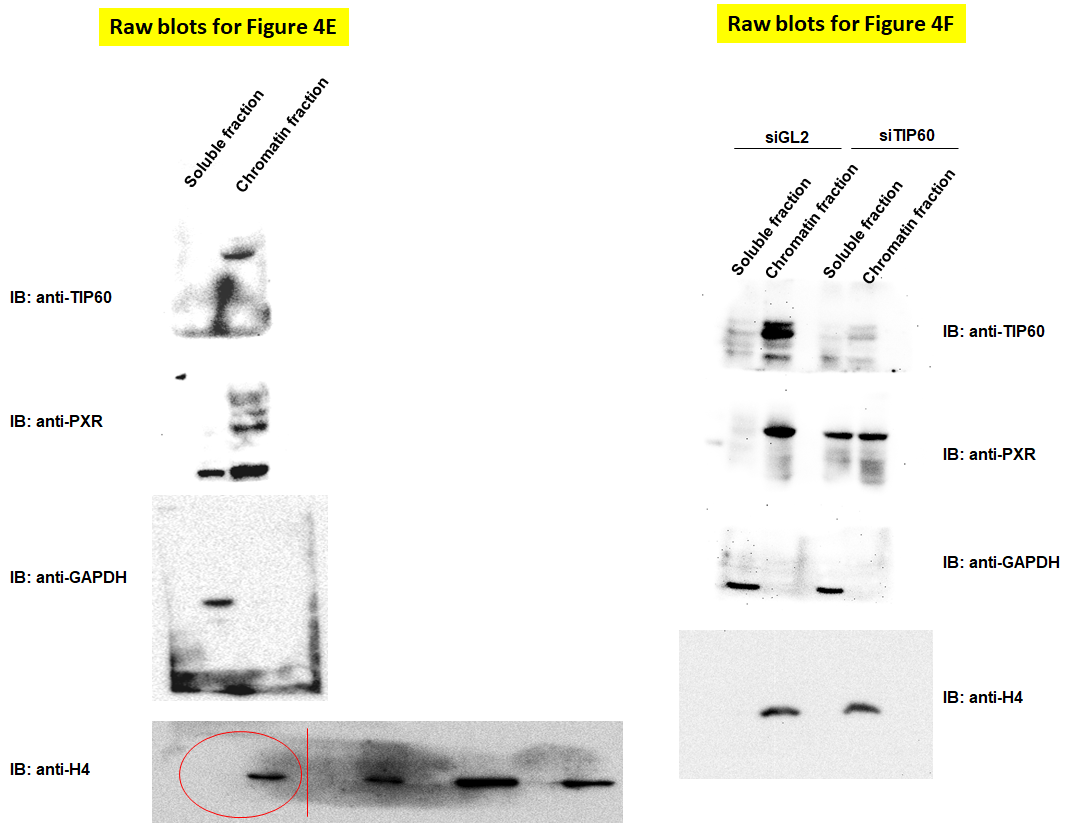


***(G) Raw images for Figure 5A & 5B***


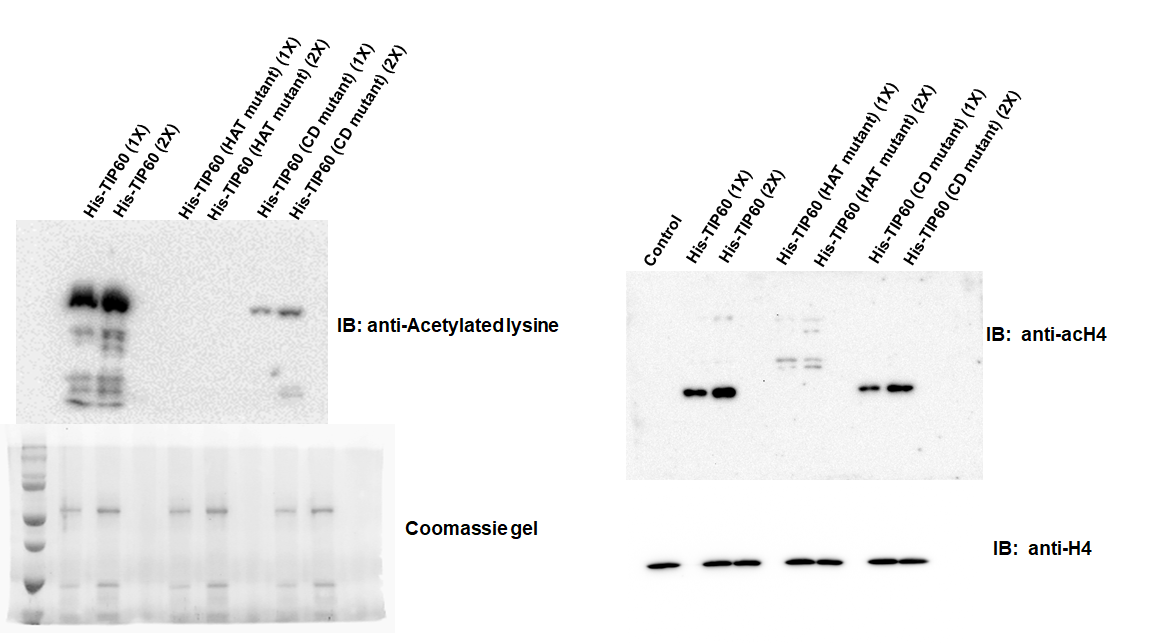


**Supporting Table 1**

| **S.No.** | **Gene** | **Gene ID** | **Accession no.** |
| --- | --- | --- | --- |
|  | *Cdc42* | 998 | NM_001791.4 |
|  | *ROCK1* | 6093 | NM_005406.3 |
|  | *IGFBP-1* | 3484 | NM_000596.4 |
|  | *Gadd45β* | 4616 | NM_015675.4 |
|  | *FGF-2* | 2247 | NM_002006.5 |
|  | *Snail* | 6615 | NM_005985.4 |
|  | *Paxillin* | 5829 | NM_002859.4 |
|  | *N-cadherin* | 1000 | NM_001792.5 |
|  | *p21* | 1026 | NM_000389.5 |
|  | *GAPDH* | 2597 | NM_002046.7 |
|  | *TGF-β* | 7040 | NM_000660.7 |
|  | *PEPCK1* | 5105 | NM_002591.4 |
|  | *Rac1* | 5879 | NM_006908.5 |
|  | *RhoA* | 387 | NM_001664.4 |
|  | *Vimentin* | 7431 | NM_003380.5 |
|  | *Fibronectin* | 2335 | NM_212482.4 |
|  | *Cofilin* | 1072 | NM_005507.3 |
|  | *β-actin* | 60 | NM_001101.5 |
|  | *28S* rRNA | 100008589 | NR_003287.4 |

**Supporting Table 2: List of primers used in the study**

| **S.**  **No.** | **Primer** | **Sequence (5’-3’)** |
| --- | --- | --- |
| 1 | hTIP60-KpnI-Fw | GG**GGTACC**ATGGCGGAGGTGGGGGAG |
| 2 | hTIP60-BamHI-Rv | CG**GGATCC**TCACCACTTCCCCCTCTTGC |
| 3 | hPXRNterm-EcoRI Fw | CG**GAATTC**ATGCTGGAGGTGAGACCCA |
| 4 | hPXRLBD-BamHI Rv | CG**GGATCC**TCAGCTACCTGTGATGCCG |
| 5 | hTIP60-Chromodomain double mutant Fw | AGTGGCCGGAAGCTTGCCTACGTCCATGCCATTGACTTCAAC |
| 6 | hTIP60-Chromodomain double mutant Rv | TTTGTTGAAGTCAATGGCATGGACGTAGGCAAGCTTCCGGCC |
| 7 | hPXR-DBD deletion mutant Fw | GAGGAAGTCGGAGGTCCCGAACGGACAGGGACT |
| 8 | hPXR-DBD deletion mutant Rv | AGTCCCTGTCCGTTCGGGACCTCCGACTTC |
| 9 | hTIP60-BamHI-Fw | CG**GGATCC**ATGGCGGAGGTGGGGGAG |
| 10 | hTIP60- EcoRI-Rv | CG**GAATTC**TCACCACTTCCCCCTCTTGCT |
| 11 | RT-PEPCK1 Fw | GATCATCTCCTTTGGCAGTGG |
| 12 | RT-PEPCK1 Rv | ATACCCAGAATCAGCATGTGC |
| 13 | RT-GAPDH Fw | ACTGCTGGGGAGTCCCTG |
| 14 | RT-GAPDH Rv | AACTGGTTGAGCACAGGGTA |
| 15 | RT-ROCK1 Fw | CTGTAACCCAAGGAGATGTG |
| 16 | RT-ROCK1 Rv | CACAATTGGCAGGAAAGTGG |
| 17 | RT-p21 Fw | TGGAACTTCGACTTTGTCAC |
| 18 | RT-p21 Rv | CACATGGTCTTCCTCTGCT |
| 19 | RT-GADD45β Fw | CGGTGGAGGAGCTTTTGGT |
| 20 | RT-GADD45β Rv | GCAGAAGGACTGGATGAGC |
| 21 | RT-Fibronectin Fw | TGATCACATGGACGCCTGC |
| 22 | RT-Fibronectin Rv | GTGGAGACAATGGTGTCACC |
| 23 | RT-FGF2 Fw | GCTGTACTGCAAAAACGGGG |
| 24 | RT-FGF2 Rv | ATCCGTAACACATTTAGAAGCC |
| 25 | RT-RhoA Fw | CAGAAAAGTGGACCCCAGAA |
| 26 | RT-RhoA Rv | GCTGCTCTCGTAGCCATTTC |
| 27 | RT-Cdc42 Fw | GTGTGTTGTTGTGGGCGATG |
| 28 | RT-Cdc42 Rv | TGTGGATAACTCAGCGGTCG |
| 29 | RT- IGFBP-1 Fw | CTCGTGCTCGGAGGTCACC |
| 30 | RT- IGFBP-1 Rv | AGCATGGGGAGCGGAGGCG |
| 31 | RT-Cofilin Fw | TCTTCTGCCTGAGTGAGGAC |
| 32 | RT-Cofilin Rv | CTCCTTCTTGCTCTCCTTGG |
| 33 | RT-Paxillin Fw | ATTCGTGAACGGCAGCTTCT |
| 34 | RT-Paxillin Rv | CTTGAGGCAGAAGGCACAG |
| 35 | RT-Vimentin Fw | GGACTCGGTGGACTTCTCG |
| 36 | RT-Vimentin Rv | AGGCGCGACTTGCCTTGG |
| 37 | RT-N-cadherin Fw | CAGGTTTGGAATGGGACAGT |
| 38 | RT-N-cadherin Rv | CGATCAAGTCCAGCTGCCA |
| 39 | RT-Snail Fw | TTCTCTAGGCCCTGGCTGC |
| 40 | RT-Snail Rv | TTGTGGAGCAGGGACATTCG |
| 41 | RT-TGFβ Fw | ACCAACTATTGCTTCAGCTCC |
| 42 | RT-TGFβ Rv | TACAGGGCCAGGACCTTGC |
| 43 | RT-Rac1 Fw | GGAGACGGAGCTGTAGGTAA |
| 44 | RT-Rac1 Rv | ACACATCTGTTTGCGCATAGG |
| 45 | RT-β-actin Fw | GCGTGGCTACAGCTTCACC |
| 46 | RT-β-actin Rv | AAGAGTGCCTCAGGGCAGC |
| 47 | RT-28S rRNA Fw | GGTGCAGATCTTGGTGGTAG |
| 48 | RT-28S rRNA Rv | GACTCCCTTTCGATCGGCC |
| 49 | Ch-ROCK1 (-10to-9)Fw | TGGTCCAGGTGATAACATCTAT |
| 50 | Ch- ROCK1 (-10to-9)Rv | AGCTTGCAGTGAGCCGATATT |
| 51 | Ch- ROCK1 (-9to-8)Fw | CCGCCTCCCGGGTTCAAG |
| 52 | Ch- ROCK1 (-9to-8)Rv | AGTAGTTGCATGCCTTCTTATTT |
| 53 | Ch- ROCK1 (-8to-7)Fw | AATAGTGGGAGCATAAACTTGTA |
| 54 | Ch- ROCK1 (-8to-7)Rv | TTTGAAACCTGCATAATATTCCAT |
| 55 | Ch- ROCK1 (-7to-6)Fw | GTTACAATTAGAGTAAATATAAGAC |
| 56 | Ch- ROCK1 (-7to-6)Rv | AACTCACAACCCCGAGCATG |
| 57 | Ch- ROCK1 (-6to-5)Fw | GATAACAGTAGTGTTATCAAAATG |
| 58 | Ch- ROCK1 (-6to-5)Rv | TGCCCACAATAGACCCTTGG |
| 59 | Ch- ROCK1 (-5to-4)Fw | GCTTCCTTTCATAGTGGGTAAT |
| 60 | Ch- ROCK1 (-5to-4)Rv | ATGGCTGCAGTGAGCTAGGA |
| 61 | Ch- ROCK1 (-4to-3)Fw | GAACTTGTGGGCTGAAGCAAT |
| 62 | Ch- ROCK1 (-4to-3)Rv | ACTCCCCCATTCATAACTATGA |
| 63 | Ch- ROCK1 (-3to-2)Fw | TCTGTAGTTAGATTGTCTCTGT |
| 64 | Ch- ROCK1 (-3to-2)Rv | GTGTTTTGTTTGTTTTATTTTGTTTT |
| 65 | Ch- ROCK1 (-2to -1)Fw | CAAAAATTAGCTGGGCGTGGT |
| 66 | Ch- ROCK1 (-2to-1)Rv | CAAACTTTATTTTTGCTCAGAAGC |
| 67 | Ch- ROCK1 (-1to0)Fw | ATGAGGAATAAATTAAGGTAGATTT |
| 68 | Ch- ROCK1 (-1to 0)Rv | AATATGTCCGCCTTCCTGTTC |
